# Cerebral Metabolism Is More Susceptible to Oxythiamine Exposure Compared to Cardiac Metabolism

**DOI:** 10.3390/ijms27209000

**Published:** 2026-10-11

**Authors:** Artem V. Artiukhov, Anastasia V. Graf, Dmitry V. Chistyakov, Olga N. Solovjeva, Vasily A. Ivlev, Nadejda R. Borisova, Vasiliy G. Vasil’ev, Maria V. Maslova, Marina G. Sergeeva, Vasily A. Aleshin

**Affiliations:** 1 A. N. Belozersky Institute of Physicochemical Biology, Lomonosov Moscow State University, 119234 Moscow, Russia; 2 Department of Biochemistry, Sechenov University, 105043 Moscow, Russia; 3 Department of Biology, Lomonosov Moscow State University, 119234 Moscow, Russia; 4 Institute of Pharmacy and Biotechnology, Peoples’ Friendship University of Russia (RUDN University), 117198 Moscow, Russia

**Keywords:** oxythiamine, thiamine, vitamin B1, pyruvate dehydrogenase, 2-oxoglutarate dehydrogenase, transketolase, NMR metabolomics, 3-hydroxybutyrate, glutathione

## Abstract

Thiamine (vitamin B1) deficiency is known to cause predominantly neurological and cardiovascular symptoms due to the metabolic function of its diphosphate derivative as a coenzyme. A variety of natural and synthetic thiamine antimetabolites can simulate this deficiency. Oxythiamine may be spontaneously produced from thiamine itself and is known to accumulate in patients with kidney disorders. In this study, tissue specificity of chronic oxythiamine administration was investigated in male rats, focusing on changes in the physiology and thiamine-dependent enzyme activities of the cerebral cortex and heart. While oxythiamine treatment significantly reduced the pyruvate dehydrogenase activity in both tissues, its cerebral effects also included mild downregulation of another thiamine-dependent enzyme, 2-oxoglutarate dehydrogenase, indicating higher susceptibility of the cerebral metabolism to oxythiamine exposure. The oxythiamine neurological effects were also reflected in the rats’ altered anxiety-like behavior and heart rate regulation. Further investigation of the cerebral effects of oxythiamine identified elevated 3-hydroxybutyrate and protein acetylation as indicators of perturbed acetyl-CoA utilization in the TCA cycle. Some heart-specific changes, such as decreased glutathione level, were also observed upon oxythiamine treatment. The present results highlight the consequences of chronic oxythiamine exposure. These finding are potentially relevant for understanding the metabolic changes in the brain and heart upon pathophysiological oxythiamine accumulation.

## 1. Introduction

Thiamine (vitamin B1) serves as a precursor of coenzyme thiamine diphosphate (ThDP), linking the metabolism of carbohydrates, amino acids, and lipids [1]. The family of ThDP-dependent enzymes in humans includes three enzymes of central metabolism, namely, pyruvate dehydrogenase complex (PDHC), catalyzing oxidative decarboxylation of pyruvate to acetyl-CoA; 2-oxoglutarate dehydrogenase complex (OGDHC), producing succinyl-CoA from 2-oxoglutarate in a similar reaction; and transketolase (TKT), an enzyme of pentose phosphate shunt, generating ribose-5-phosphate and NADPH from glucose for various biosynthetic reactions. Other less known mammalian ThDP-dependent enzymes are associated with peripheral metabolism. These include branched-chain 2-oxo acid dehydrogenase complex, involved in degradation of valine, leucine, isoleucine, threonine, and methionine; 2-oxoadipate dehydrogenase complex—OGDHC isozyme decarboxylating 2-oxoadipate, an intermediate in lysine and tryptophan degradation; and 2-hydroxyacyl-CoA lyase, participating in fatty acid alpha-oxidation [2,3,4,5,6].

The effects of thiamine deficiency were first described before the coenzyme role of ThDP was elucidated [7] and, since then, they have been observed in the nervous system as neuropsychiatric symptoms, known as Wernicke–Korsakoff syndrome in case of compromised nutrition, mostly associated with alcoholism [4,8,9]. Although thiamine deficiency in the brain develops slower than in other tissues [10], neurological symptoms were considered the first clinical signs of the deficiency. This is followed by growth depression [11] and dysfunctions of other organs, including cardiovascular, respiratory, gastrointestinal, and musculoskeletal systems [12]. Among those, cardiological symptoms are often described after neurological ones, such as in the case of “wet beriberi” and “Shoshin beriberi” diseases, associated with tachycardia, increased cardiac chambers, and heart failure rather than neuropathy [13]. Thus, the brain and heart seem particularly sensitive to thiamine deficiency.

**Figure 1:**
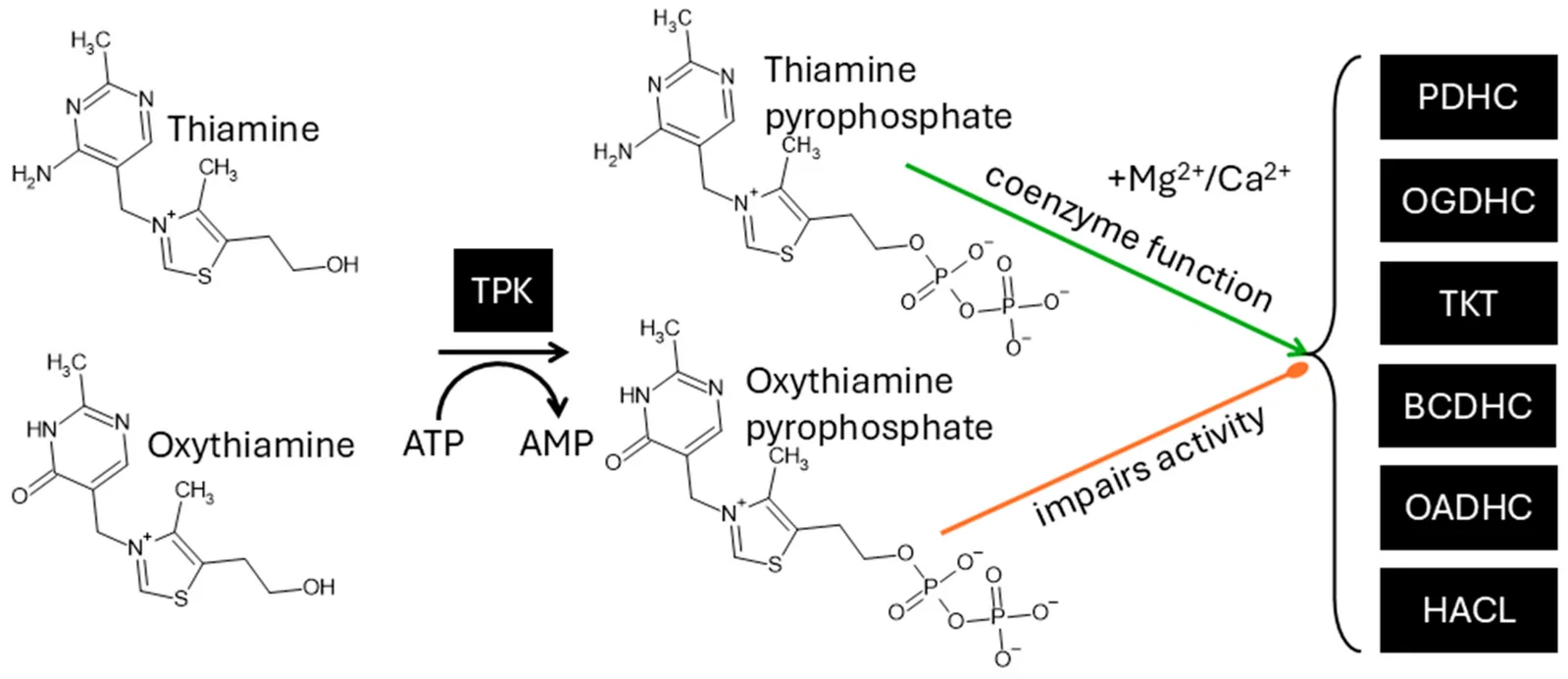
Structures of thiamine, oxythiamine, and their diphosphate (pyrophosphate) derivatives, acting on ThDP-dependent enzymes. TPK—thiamine pyrophosphokinase, PDHC—pyruvate dehydrogenase complex, OGDHC—2-oxoglutarate dehydrogenase complex, TKT—transketolase, BCDHC—branched-chain 2-oxo acid dehydrogenase complex, OADHC—2-oxoadipate dehydrogenase complex; HACL—2-hydroxyacyl-CoA lyase.

Although models of thiamine deficiency can rely on “thiamine-free” conditions [10,14], the ubiquity of thiamine makes such conditions rather difficult to reproduce. Thus, different thiamine antagonists are widely used, providing several mechanisms for studying thiamine deficiency [15,16]. For example, administration of pyrithiamine, an inhibitor of thiamine pyrophosphokinase which phosphorylates thiamine to ThDP (Figure 1), is known to result in fast and severe neurologic symptoms of thiamine deficiency [17,18]. Other antagonists are oxythiamine, 3-deazathiamine, 2-methylthiamine, amprolium, and some less obvious ones such as antidiabetic metformin and the antibiotics metronidazole and bacimethrin [2,15,16,19,20]. Among those, oxythiamine (Figure 1) takes a unique place in human metabolism, because together with its phosphorylated derivatives, oxythiamine is constantly present at low amounts even in healthy subjects [21]. It is thought to be spontaneously produced from thiamine during food heating [21] and, reportedly, via enzymatic oxidation in some bacteria [22]. Various syndromes are known to elevate oxythiamine and oxythiamine diphosphate (oThDP, Figure 1) in humans and mammals, including renal deficiency [21,23]. Thus, oxythiamine can be considered a naturally occurring thiamine derivative, which accumulates under certain pathological conditions, and thus, unlike other thiamine antagonists, it is a more physiologically relevant model of thiamine deficiency. Previous studies have demonstrated that oxythiamine administration to animals results in biochemical and physiological manifestations similar to thiamine deficiency [11,15,24,25,26]. These manifestations predominantly occur through the action of oThDP, which competes with coenzyme ThDP for the active sites of ThDP-dependent enzymes (Figure 1), although a non-coenzyme action cannot be excluded completely [16,27,28,29].

While the effects of thiamine antagonists, including oxythiamine, mainly result from the inhibition of the mentioned ThDP-dependent enzymes, their in vivo effects are often specific for a cell line, tissue, or organ [19,30]. Recently, we demonstrated that excessive thiamine itself affects the enzymes in the rat cerebral cortex and heart more than in other tissues [31]. Here, we aim at characterization of these two main thiamine-requiring tissues upon chronic administration of a naturally occurring thiamine antagonist, oxythiamine. This should contribute to a better understanding of the mechanisms underlying neurologic and cardiologic symptoms conventionally associated with thiamine deficiency and pathophysiological oxythiamine accumulation occurring, for example, in patients upon renal failure [21].

## 2. Results

### 2.1. Oxythiamine Treatment Reduces Blood ThDP Level and Transketolase Activity

**Figure 2:**
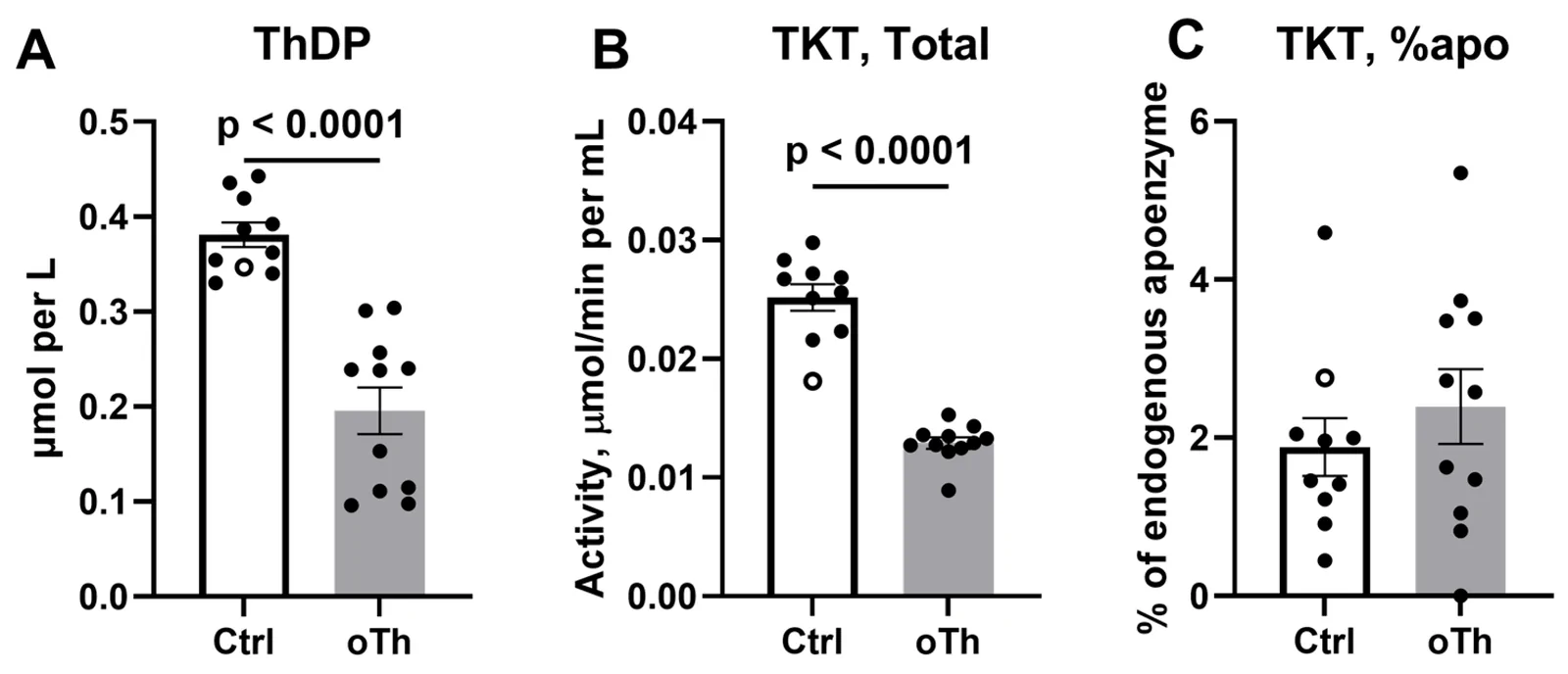
Changes in thiamine diphosphate (ThDP) concentration (**A**), transketolase (TKT) total activity in the presence of external ThDP (**B**), and percentage of TKT activation by external ThDP, i.e., fraction of endogenous TKT apoenzyme (**C**), in the whole blood of rats upon chronic oxythiamine (oTh) treatment (n = 11), compared to the control (Ctrl) rats (n = 10). Significant (*p* ≤ 0.05) effects evaluated using *t*-test are shown as *p*-values above the graphs. Hollow circles correspond to the animal excluded from the multivariate analyses (see Section 4.6).

Evaluation of blood ThDP levels and analysis of TKT activity are useful markers for the estimation of overall ThDP content. They are especially relevant with regard to the focus of this study on the heart and the brain metabolism and known resistance of these organs to developing thiamine deficiency [10]. In this study, blood ThDP concentration was reduced twice in the oxythiamine-treated rats, compared to the control ones (Figure 2A). Total blood TKT activity was also halved by oxythiamine supplementation (Figure 2B). However, TKT activity is similarly decreased both with and without external ThDP, resulting in almost no enzyme fraction present in apoenzyme form (Figure 2C). The latter differentiates TKT of oxythiamine-treated animals from nutritionally thiamine deficient patients [32]. Thus, the observed two-fold reduction in ThDP and TKT activity upon initial screening of thiamine markers in the blood demonstrates the development of a substantial effect of oxythiamine treatment at the level of the whole organism. This enables further focus on studying the metabolism of the heart and the brain as the most thiamine-dependent organs.

### 2.2. Oxythiamine Treatment Affects the Brain Rather than Heart Physiology

**Figure 3:**
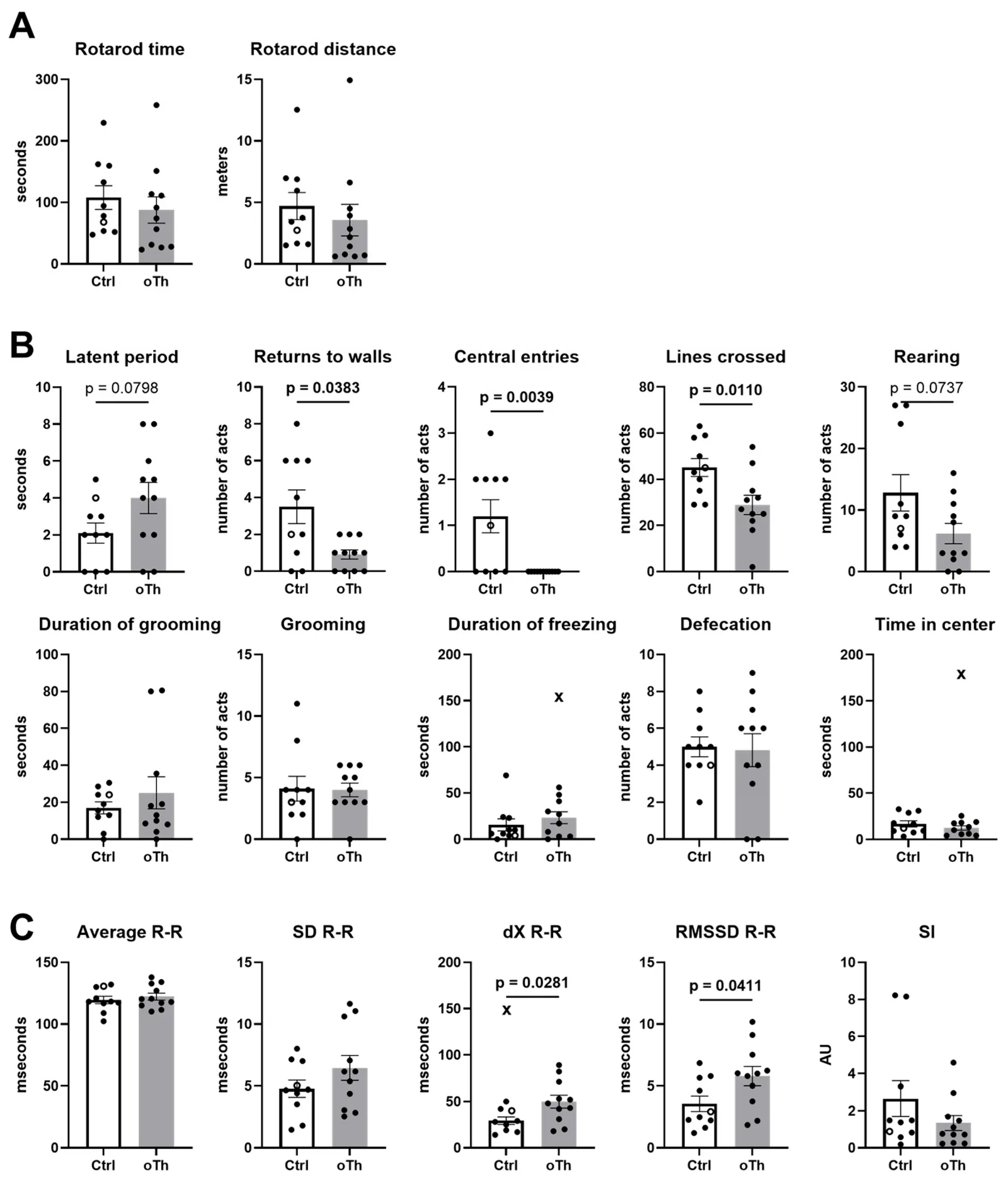
Changes in rat physiology upon 29 days of chronic oxythiamine (oTh) treatment. (**A**) Time and distance on rotarod. (**B**) Behavioral parameters in open field test. (**C**) Heart rate and its variability parameters measured by electrocardiography (ECG); the following parameters are determined: average R-R interval, standard deviation (SD) of R-R intervals, range (dX) of R-R intervals, root mean square of successive differences (RMSSD) between R-R intervals, and stress index (SI). Significant differences (*p* ≤ 0.05) and trends (0.05 < *p* ≤ 0.1) between the control (n = 10) and oTh-treated (n = 11) groups evaluated using *t*-test or Mann–Whitney U-test are shown as *p*-values above the graphs; “X” indicate outliers excluded using iterative Grubbs’ test (see Section 4.6). Hollow circles correspond to the animal excluded from the multivariate analyses (see Section 4.6).

The physiological effects of oxythiamine-induced thiamine deficiency on the function of rat muscles, nervous systems, and heart were studied using rotarod and open field tests and assessing electrocardiography (ECG) parameters after 29 days of oxythiamine administration (Figure 3). The rotarod test showed no significant changes in either time or distance, indicating that general motor ability and muscular endurance were preserved in oxythiamine-treated animals (Figure 3A). However, voluntary locomotion in the open field test was strongly affected (Figure 3B). The observed reduction (*p* < 0.05) in the number of crossed lines, central entries, and returns to the area walls indicates the increased anxiety-like behavior of oxythiamine-treated rats, despite such indicators as grooming, freezing, and defecations not being affected significantly by the oxythiamine administration (Figure 3B). Additional trends to a longer latent period (*p* = 0.08) and decreased number of rearing acts (*p* = 0.07) further support this observation.

Among the ECG parameters, increased range (dX) and root mean square of successive differences (RMSSD) of R-R intervals were observed upon oxythiamine administration (Figure 3C), whereas the duration of R-R intervals itself was not affected. Such effects are likely to reflect increased parasympathetic activity of the autonomous nervous system, showing altered autonomic modulation of the heart.

Comparison of these physiological data to the results obtained at experimental day 16 can be made. Such a comparison reveals that the observed effects of oxythiamine on locomotory behavior and ECG regulation were not induced by only two weeks of oxythiamine supplementation (Supplementary Figure S1A,B). Instead, the oxythiamine treatment seems to have initially reduced the animals’ anxiety-like behavior, based on trends of reduced numbers of grooming acts (*p* = 0.09) (Supplementary Figure S1A). Thus, development of thiamine deficiency by the end of a month-long supplementation of oxythiamine (Figure 2) induced anxiety-like locomotory behavior and heart rate variability changes suggestive of increased parasympathetic influence in rats, not affecting endurance (Figure 3). These physiological signs of the deficiency were missing at the end of the first two weeks of the treatment, when a mild reduction in anxiety-like behavior was observed (Supplementary Figure S1A).

### 2.3. Oxythiamine Effects on the Essential ThDP-Dependent Enzymes in the Heart and Cerebral Cortex

**Figure 4:**
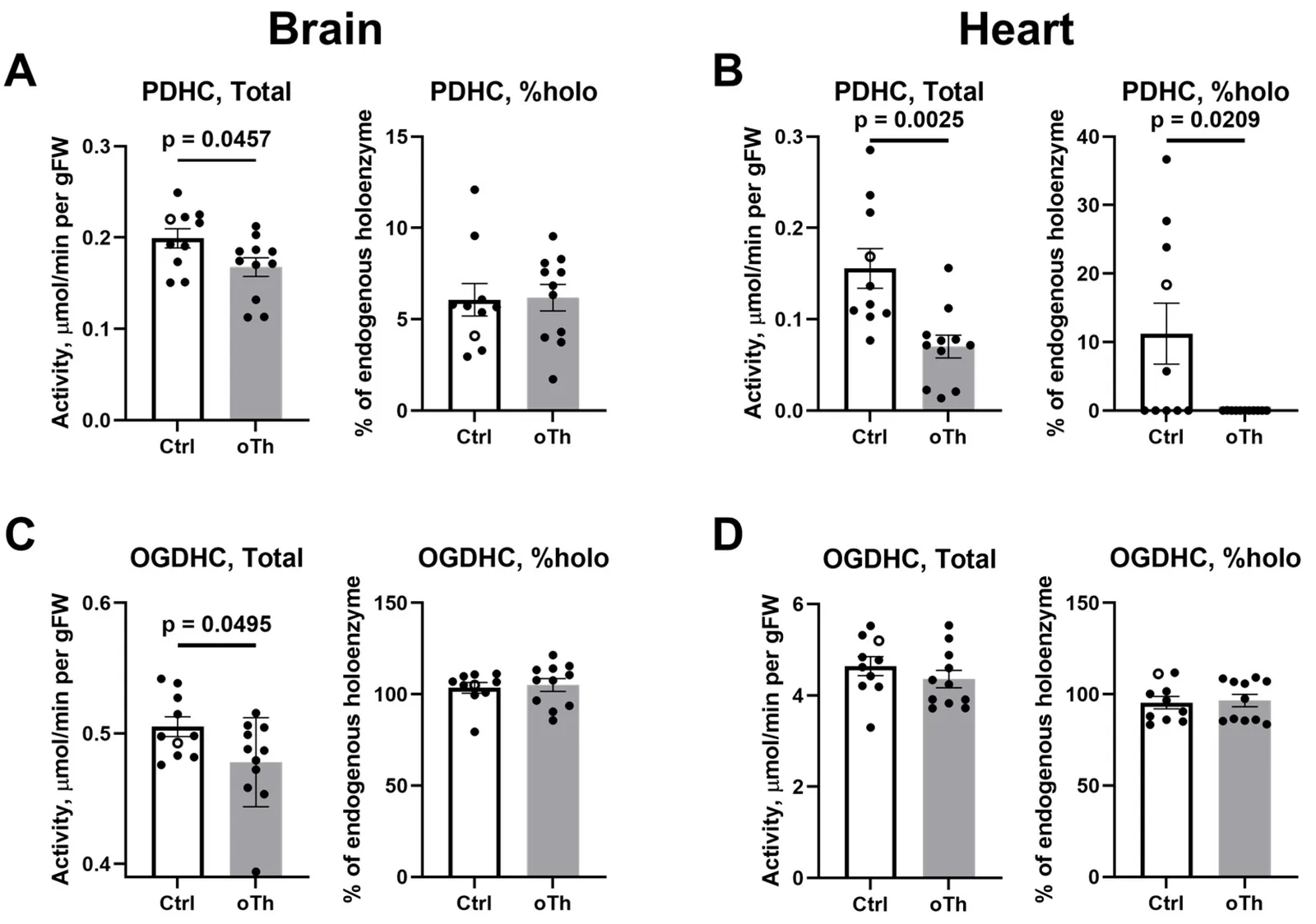
Changes in the activities of rat thiamine diphosphate (ThDP)-dependent enzymes in rat cerebral cortex (**A**,**C**) and heart (**B**,**D**) upon chronic oxythiamine (oTh) treatment. Pyruvate dehydrogenase complex (PDHC, (**A**,**B**)) and 2-oxoglutarate dehydrogenase complex (OGDHC, (**C**,**D**)) were assayed fluorometrically in the medium with and without ThDP per g of tissue fresh weight (gFW). Endogenous holoenzyme fractions were calculated as ratios between the activities without and with ThDP added to the medium. Significant differences (*p* ≤ 0.05) between the control (n = 10) and oTh-treated (n = 11) groups evaluated using *t*-test or Mann–Whitney U-test are shown as *p*-values above the graphs. Hollow circles correspond to the animal excluded from the multivariate analyses (see Section 4.6).

Since the effects of oxythiamine treatment were observed as changes in both CNS (anxiety-like behavior) and heart (ECG variability) parameters, we further determined which of these had the stronger changes in the key thiamine-dependent enzymes PDHC and OGDHC. The total PDHC activity (i.e., assayed in the presence of saturating ThDP concentration) decreased both in the cerebral cortex (Figure 4A) and heart (Figure 4B) of oxythiamine-treated rats. In addition, none of the oxythiamine-treated animals demonstrated detectable cardiac PDHC holoenzyme activity (assayed in the absence of added ThDP), resulting in a significant decrease compared to control rats (Figure 4B). No such reduction was observed in the cerebral cortex (Figure 4A). However, one should take into account that the endogenous PDHC holoenzyme activity was usually low, about 5–10% of the total activity (Figure 4), which is fairly close to the assay detection limit, sometimes resulting in zero activity [33].

OGDHC, another ThDP-dependent enzyme, has a much stronger affinity towards ThDP [2]; thus, even a mild effect of oxythiamine on OGDHC activity reveals signs of functional thiamine deficiency. Indeed, a marginal reduction in OGDHC activity was induced by oxythiamine administration in the cerebral cortex (Figure 4C), but not in the heart (Figure 4D). Thus, alterations in OGDHC in the cerebral cortex and not in the heart may indicate the higher sensitivity of the former tissue to the oxythiamine administration. The activities determined with and without additional ThDP were equal, resulting in both organs in app. 100% fraction of endogenous holoenzyme, not altered by oxythiamine administration (Figure 4C,D).

### 2.4. Changes in the Cerebral Cortex Metabolomics Reveal Consequences of Oxythiamine Action

While oxythiamine-induced changes in activities of the main ThDP-dependent enzymes of the brain cortex and heart were largely similar (Figure 4), physiological parameters mostly reflect the effects on the nervous system due to altered anxiety-related locomotion and ECG variability indicators (Figure 3B,C) rather than muscles or heart, as endurance in the rotarod test and R-R intervals were unchanged (Figure 3A,C). Thus, we decided to further investigate potential oxythiamine-induced metabolic changes of the brain cortex more deeply, as these changes may mediate the physiological response.

**Figure 5:**
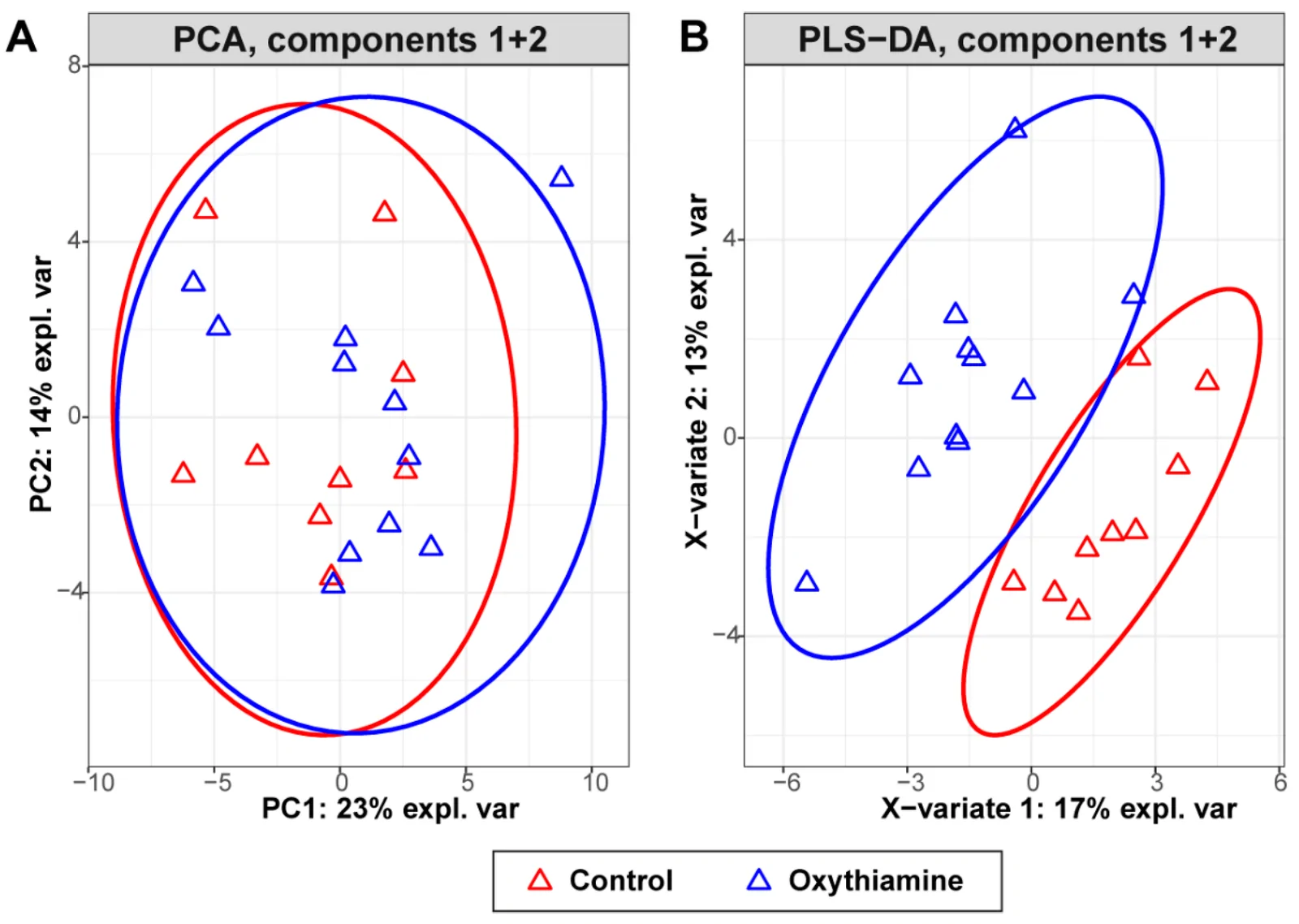
Exploratory principal component analysis (PCA, (**A**)) and partial least squares discriminant analysis (PLS-DA, (**B**)) score plots. The graphs depict two components with the highest explained variance (PC1 and PC2, (**A**)) or highest discriminating power (X-variates 1 and 2, (**B**)). Red and blue triangles indicate samples from control (n = 9) and oxythiamine-treated (n = 11) animals, respectively. Data of one control rat was excluded due to technical reasons (see Section 4.6). The 95% confidence intervals are shown with red and blue ellipses, correspondingly.

Targeted metabolomics analysis using NMR in the cerebral cortex extracts resulted in quantification of 46 metabolites. The latter included 18 proteinogenic amino acids, nine amino acid derivatives, six organic acids, four hydroxy acids, four nucleotides or their pyrimidine base precursors, three vitamins and derivatives, as well as glucose and myo-inositol (Supplementary Table S1). All these were added with quantification of reduced and oxidized glutathione, whose ratio is known to be regulated by ThDP-dependent enzymes and specific inhibitors of PDHC and OGDHC [34,35]. Levels of the main mitochondrial protein deacetylase sirtuin 3 (SIRT3) and desuccinylase sirtuin 5 (SIRT5) tightly linked to PDHC and OGDHC as producers of acetyl-CoA and succinyl-CoA, respectively, were evaluated by immunoblotting, as well as acetylation of histone and non-histone protein fractions, previously affected by the directed downregulation of PDHC [35,36]. Together with the activities of PDHC, OGDHC, and thiamine-regulated enzymes malate and glutamate dehydrogenases [16,37], the resulting dataset was analyzed by multivariate approaches, such as principal component analysis (PCA) and partial least squares discriminant analysis (PLS-DA) (Figure 5), in order to determine whether the cerebral metabolism of oxythiamine-treated animals differed from those of control ones and which parameters discriminated the two groups the most.

Unsupervised PCA approach with all metabolites and potential indicators of oxythiamine effect did not reveal clear separation between control and oxythiamine-treated samples (Figure 5A). Although the fitted PLS-DA score plot showed apparent visual separation between the groups (Figure 5B), this in-sample pattern also has limited internal predictive support. Repeated stratified three-fold cross-validation of the six-component model yielded a minimal balanced error rate of 0.368, corresponding to only 63.2% accuracy, while a permutation test with 1000 labels was not significant (*p* = 0.14). Thus, our PLS-DA model should not be interpreted as a valid classifier and metabolites with high variable importance in projection (VIP) scores cannot be used as reliable biomarkers. Nevertheless, the score plot (Figure 5B) and VIP rankings (Table 1) can still be treated as exploration of the features associated with downregulation of ThDP-dependent enzymes. The selected features showed high stability, i.e., they maintained high VIP scores, when the PLS-DA model was repeatedly cross-validated using the aforementioned three-fold partition (Table 1).

**Figure 6:**
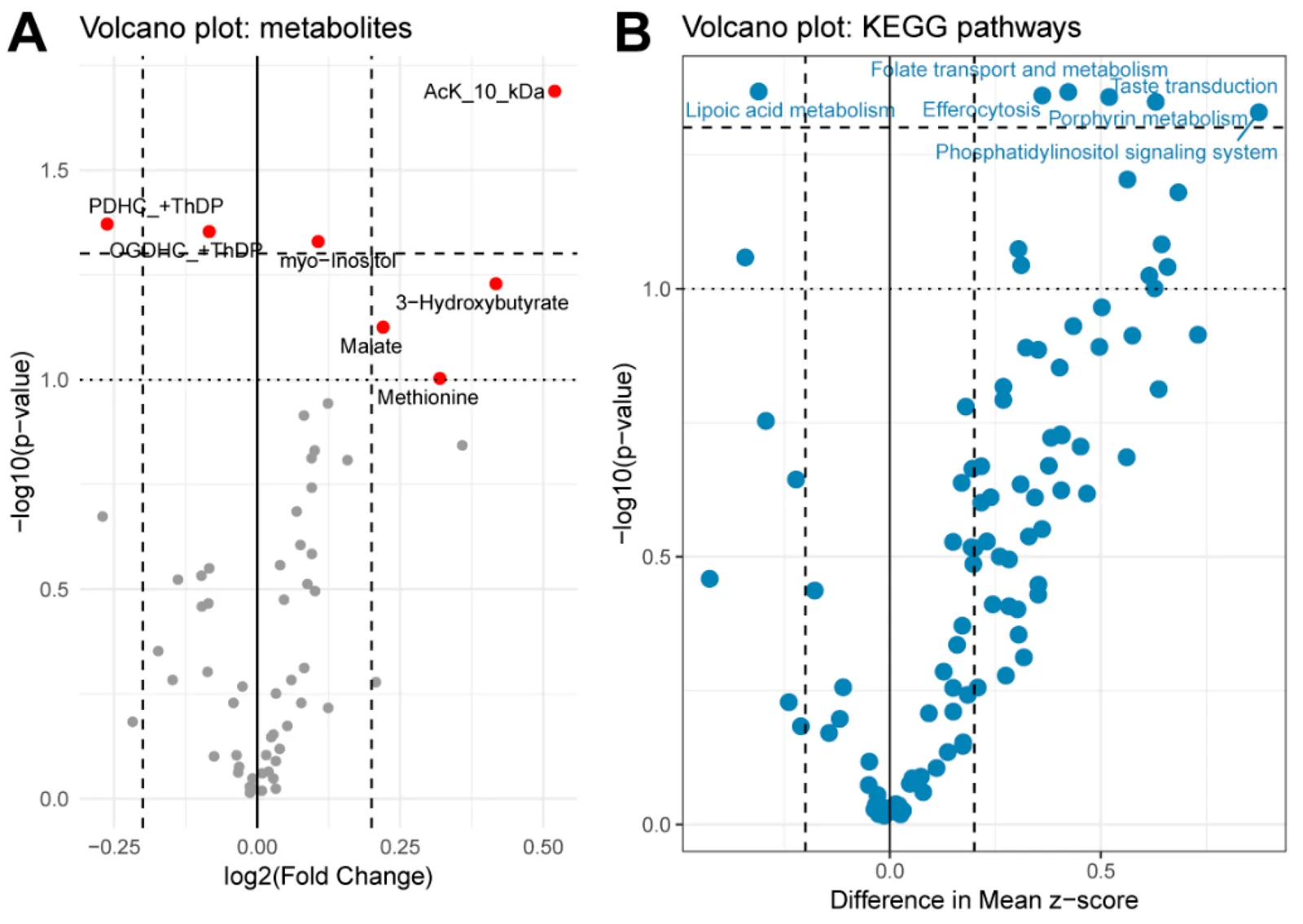
Volcano plots for all biochemical changes in the rat cerebral cortex upon oxythiamine treatment. (**A**) All biochemical parameters separately. (**B**) Parameters grouped according to KEGG pathways. Metabolites and groups which are significantly different (*p* ≤ 0.05; indicated by dashed horizontal lines) or show trends to a significant difference (0.05 < *p* ≤ 0.1; indicated by dotted horizontal lines) in oxythiamine-treated vs. control animals are labelled. Dashed and solid vertical lines indicate fold change of 1.15 and 1.00, respectively.

The most notably discriminating parameters with VIP score > 1.5 for the first PLS component included already established ThDP-dependent indicators, such as total OGDHC and PDHC activities, as well as levels of total acetylation of low-molecular-weight proteins, mainly histones (AcK 10 kDa) and several metabolites, 3-hydroxybutyrate, myo-inositol, malate, glutamate, O-phosphocholine, and methionine (Table 1 and Figure 6A). While the metabolic parameters all together do not allow visual separation of oxythiamine-treated and control animals in the clustered image map (Supplementary Figure S2A), the two groups were almost separated when the map was constructed with the filtered features (VIP score > 1.5), with only one oxythiamine-treated sample being located in the cluster of control samples (Supplementary Figure S2B). The oxythiamine-treated cluster was characterized by lower OGDHC and PDHC activities, and higher levels of other features with VIP scores above 1.5, indicating metabolic rearrangement underlying altered ThDP-dependent pathways (Table 1 and Figure 6A).

Thus, the features that discriminate oxythiamine-treated animals can be classified as (1) lower total PDHC and OGDHC activities; (2) higher levels of protein acetylation and 3-hydroxybutyrate; (3) higher glutamate upon altered TCA cycle mediated by OGDHC; (4) higher malate and methionine associated with decreased oxidation of pyruvate and 2-oxobutyrate, respectively, via PDHC; and, finally, (5) elevated myo-inositol indicating a downregulated pentose phosphate pathway mediated by TKT.

In order to better characterize the functional significance of the metabolic changes (Figure 6A), the biochemical parameters were grouped according to KEGG pathways annotation (Figure 6B). The significantly (*p* ≤ 0.05) downregulated pathways included only “Lipoic acid metabolism”, comprising PDHC and OGDHC activities as well as levels of their substrate, NAD^+^, branched-chain amino acids, and glycine. The most significantly upregulated pathways included (1) “Folate transport and metabolism”, comprising methionine and its derivatives; (2) “Efferocytosis”, comprising methionine, lactate and NAD^+^; as well as less specific (3) “Taste transduction” and (4) “Porphyrin metabolism” comprising multiple TCA cycle intermediates like citrate and TCA-cycle coupled amino acids, like glutamate and glutamine (Figure 6B). The latter is in accordance with PDHC and OGDHC downregulation induced by oxythiamine.

### 2.5. Heart-Specific Reduction in Glutathione Level Indicates Redox Stress

**Figure 7:**
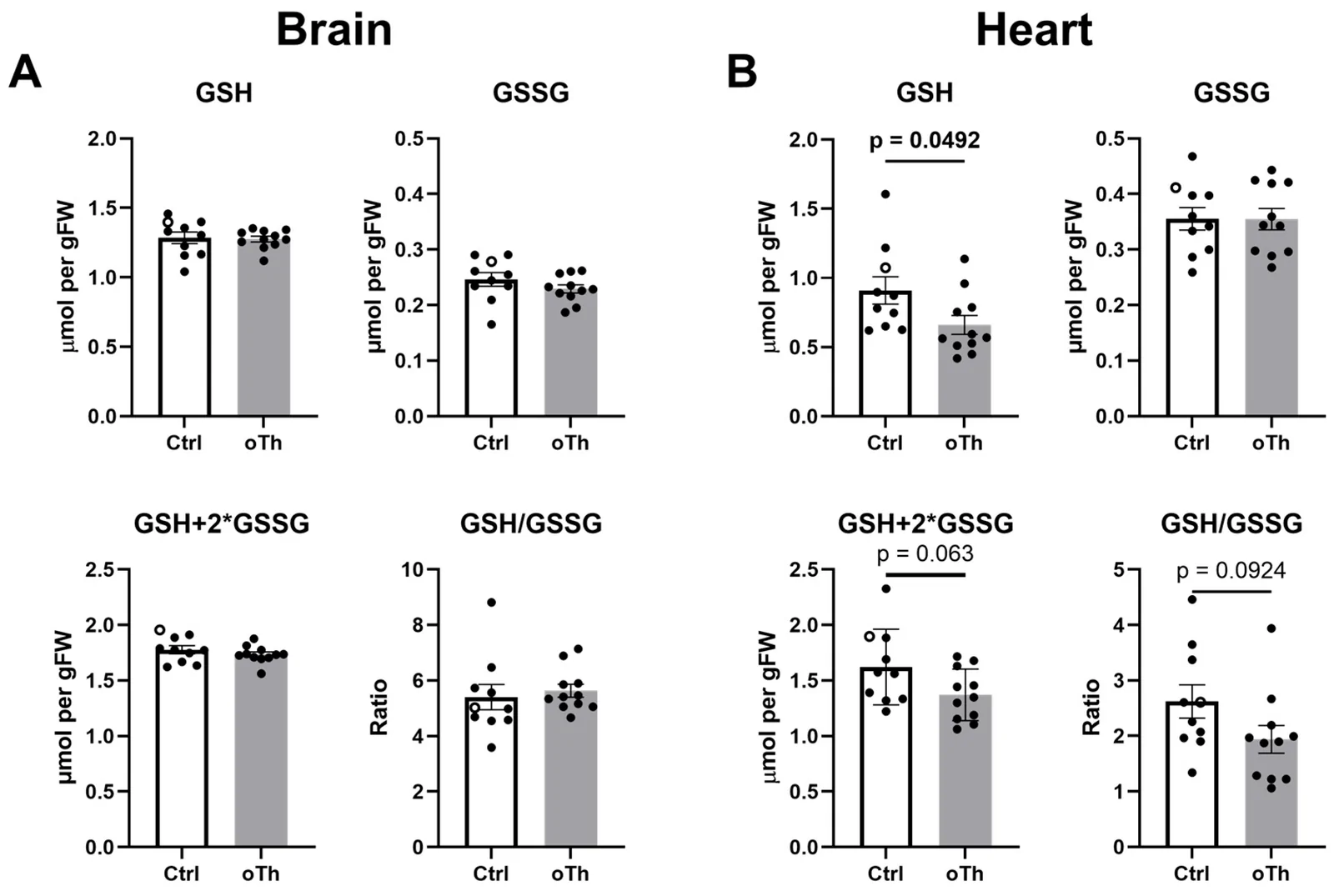
Changes in the glutathione redox potential in the rat cerebral cortex (**A**) and heart (**B**) upon chronic oxythiamine (oTh) treatment. Significant differences (*p* ≤ 0.05) between the control (n = 10) and oTh-treated (n = 11) groups evaluated using *t*-test are shown as *p*-values above the graphs. GSH—glutathione, GSSG—glutathione disulfide, GSH+2*GSSG—total glutathione, i.e., sum of GSH and double GSSG amounts, gFW—g of tissue fresh weight. Hollow circles correspond to the animal excluded from the multivariate analyses (see Section 4.6).

Multiple metabolic changes in the cerebral cortex indicate changes in PDHC- and OGDHC-dependent pathways (Figure 4 and Figure 6), which may alter the redox status of the cells and tissues [34,35]. The latter is largely mediated by the ratio of reduced (GSH) and oxidized (GSSG) forms of glutathione. However, the cerebral cortex did not demonstrate any oxythiamine-induced changes in the levels of GSH, GSSG, their ratio, or the sum (Figure 7A), but the initially comparatively low cardiac GSH level was decreased further (*p* < 0.05) following oxythiamine administration (Figure 7B). The level of cardiac GSSG was preserved upon oxythiamine administration. Thus, the cerebral cortex seems to have remained more resistant to the oxythiamine-induced redox imbalance, compared to the heart. The latter may have been due to the 40% higher GSH and about 2-fold higher GSH/GSSG ratio in the cerebral cortex than in the heart, evident from the control animals’ data (Figure 7A vs. 7B). The observed decrease in cardiac GSH level indicates substantial redox stress, which also corresponds well with a strong decrease in cardiac PDHC activity upon long-term oxythiamine exposure (Figure 4B).

### 2.6. Correlations Between Metabolic Parameters and Animal Physiology

Although metabolic changes in oxythiamine group were moderate, correlation analysis may help with inter-organism variability by identifying the parameters linked with stronger physiological responses. Specifically, metabolic parameters significantly correlated with physiological ones are likely to be involved in physiological response to chronic exposure to oxythiamine, although no causative connection can be implied directly from a correlation.

**Figure 8:**
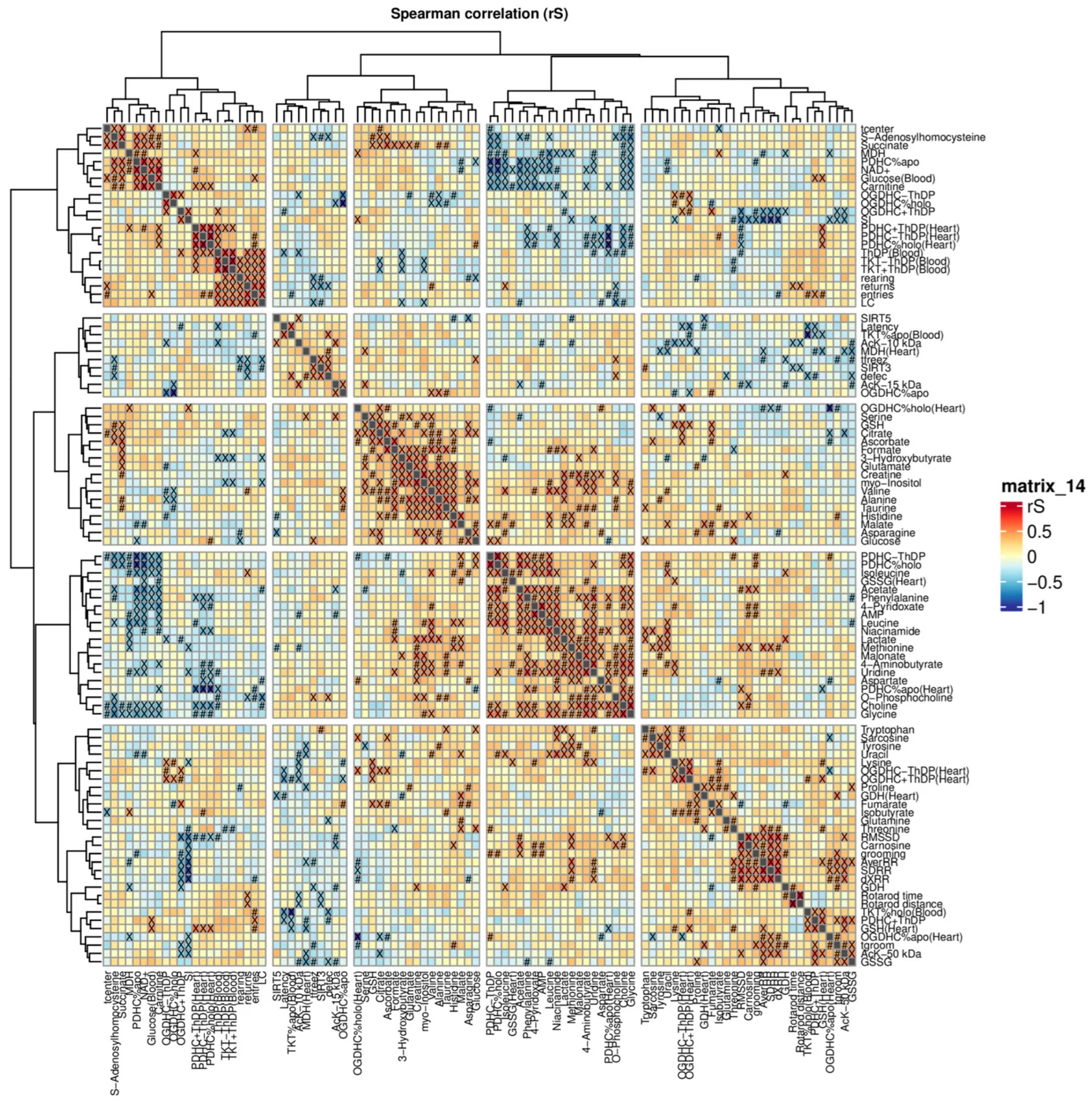
Spearman correlations of physiological vs. metabolic parameters in control and oxythiamine rats combined (n = 21). Clusters (1–5) of positively correlated parameters are outlined with black rectangles. The color scheme presented in the legend corresponds to the correlation coefficient (rS). Significant differences (*p* ≤ 0.05) and trends (0.05 < *p* ≤ 0.1) are marked with “X” and “#” signs, respectively, within the corresponding cells. The parameters determined in the heart and blood are labelled accordingly using brackets. MDH—malate dehydrogenase, GDH—glutamate dehydrogenase, tcenter—time spent in center, LC—number of line crossings, tfreez—freezing duration, defec—number of defecation acts, tgroom—grooming duration; other parameters are abbreviated in Materials and Methods and throughout the text. Activities of PDHC, OGDHC, and TKT are depicted as “−ThDP” (endogenous holoenzyme activity), “+ThDP” (total activity measured with added ThDP), “%holo” (fraction of the holoenzyme), or “%apo” (fraction of the apoenzyme).

Using correlation matrices, comprising all studied biochemical indicators and physiological parameters, we show several clusters of positively correlating parameters (Figure 8). Particularly, Cluster 1 represents key locomotory parameters in the open field test, such as numbers of rearing acts, central entries, returns to the walls, and line crossings, which were all significantly reduced by oxythiamine treatment (Figure 3). These locomotory parameters correlate with the most significantly affected ThDP-dependent indicators also included in Cluster 1 (Figure 8), such as blood TKT activity and ThDP level, cerebral OGDHC and cardiac PDHC. The latter are complemented by OGDHC and PDHC substrate NAD^+^ as well as the levels of carnitine and other energy metabolism indicators, like blood glucose and cerebral TCA intermediate succinate. Such correlations of locomotory behavior in rats and key ThDP-dependent metabolic and other energy metabolism-related parameters may indicate the importance of cerebral OGDHC for energy production in this model of chronic oxythiamine supplementation to rats.

Cluster 2 (Figure 8) includes anxiety-like parameters, such as freezing time, number of defecation acts, and latent period. Those correlate with metabolic parameters upregulated by oxythiamine (Figure 6A), including levels of cerebral 3-hydroxybutyrate, and lysine acetylation, together with deacylases SIRT3, SIRT5, and their substrate NAD^+^. The latter is tightly correlated with the levels of carnitine, in addition to other energy metabolism indicators, like blood glucose and cerebral TCA intermediate succinate.

Clusters 3 and 4 do not include any behavioral or ECG indicators, but they combine a lot of important mostly cerebral metabolic biomarkers positively correlated to each other within the cognate clusters and to some extent also between them (Figure 8). Cluster 3 includes mostly proteinogenic amino acids and associated derivatives such as creatine, taurine, and GSH, together with heart OGDHC holoenzyme fraction and cerebral glucose, myo-inositol, organic acids, and hydroxy acids, including citrate, formate, ascorbate, and 3-hydroxybutyrate. Some of these parameters were significantly elevated upon oxythiamine treatment or showed trends towards elevation (Figure 6A). Cluster 4 includes cerebral levels of more amino acids, their derivatives, and other metabolites, such as choline, and O-phosphocholine, AMP, and 4-pyridoxate, niacinamide, lactate, 4-aminobutyrate, and others, as well as cerebral PDHC activity without added ThDP (holo-PDHC) and fraction of cerebral PDHC holoenzyme. Most parameters in these two clusters were only slightly affected in the rat model of chronic oxythiamine exposure. However, they may be inversely correlated with the parameters of other clusters, e.g., Clusters 1 and 2, comprising the key parameters affected by oxythiamine and general indicators of energy metabolism (Figure 8).

Finally, Cluster 5 (Figure 8) includes ECG variability parameters, some of which are also affected by oxythiamine treatment, together with the number and duration of grooming acts, and rotarod parameters, correlating with cardiac OGDHC activity and GSH level in both tissues, and with PDHC activity in the brain only, together with some TCA-cycle-related cerebral metabolites, like glutamine and fumarate. The core of Cluster 5 is represented by the ECG variability parameters, number of grooming acts, and carnosine level, inversely correlating with ECG stress index (SI) and cerebral total OGDHC activity from Cluster 1 (Figure 8). Although the latter was determined in the cerebral cortex, its potential importance for the cardiac function may result in established correlations with the R-R interval and its variability.

Finally, the issue of whether the observed correlations (Figure 8) may be driven by the differences in the group mean values of the parameters (Figure 2, Figure 3, Figure 4 and Figure 7) was addressed. We constructed the additional correlation heatmap with the adjustment of group mean ranks. The resulting correlation matrix (Supplementary Figure S3) is fairly similar to the original, as around 80% of significant correlation are retained in the group mean-corrected heatmap. However, the clustering structure is not retained, and associations of most physiological parameters with specific ThDP-dependent enzymes become weaker. This suggests that most of the correlations represent oxythiamine-induced concomitant alterations, rather than interdependences equally observed in control and oxythiamine-treated animals.

Thus, the positive and negative correlations observed within and between the clusters, respectively, together describe the altered state of metabolic network of the rat cerebral cortex and heart, induced by oxythiamine, and its connection to animal physiology.

## 3. Discussion

### 3.1. Tissue Specificity of Thiamine-Dependent Metabolism

As mentioned before, the focus of this work on the heart and cerebral cortex is determined by the preferential dependence of these energy-requiring tissues on thiamine and ThDP-dependent enzymes. Complications of their function upon thiamine deficiency are known to result in wet or dry beriberi—the forms indicating major disruptions in the cardiovascular or nervous systems, respectively [31]. Moreover, these tissues preserve their thiamine content stronger than the other tissues, with the brain being the least thiamine-releasing organ upon thiamine deficiency [10].

The effects of chronic exposure to oxythiamine on the main ThDP-dependent metabolic enzymes were relatively similar in the rat brain and heart, although altered metabolism of nervous tissue seems to have influenced rat physiology and behavior, while muscle endurance was not affected significantly (Figure 3 and Figure 4). Such dependence of the organism’s behavior on the cerebral ThDP-dependent metabolism may explain the known strong thiamine-storing capacity of the brain upon thiamine deficiency [10]. Moreover, the transport of high-dose thiamine into the heart is much more efficient compared to the cerebral cortex, which is likely to be due to the blood–brain barrier [31]. In accordance with this, in this study, blood ThDP positively correlated with cardiac but not cerebral PDHC activity (Figure 8).

It should be noted that the open field test used was a multi-dimensional paradigm that assessed locomotor activity, exploratory drive, and emotional reactivity simultaneously. The observed reduction in line crossings, central entries, and returns to walls, together with the unchanged rotarod performance, suggests that the behavioral changes were more likely to have been related to an altered emotional or motivational state rather than to a general decline in motor functions. Nevertheless, the open field test alone cannot definitively distinguish between anxiety-like behavior, reduced exploratory motivation, and other non-specific changes in the animal’s state. Therefore, the observed behavioral changes are referred to as “anxiety-like behavior”.

Interestingly, the responses of the organism to oxythiamine and to high-dose thiamine administration, which were expected to induce opposite effects, had much in common. That is, high-dose thiamine administration induced the strongest response of ThDP-dependent metabolic network in the rat cerebral cortex, followed by the heart, with other tissues being less involved [31]. For example, thiamine administration reduced both cerebral cortex PDHC activity and phosphorylation of Ser293 residue, with cardiac PDHC being more resistant. Furthermore, the two tissues differed in the response of regulatory PDHC kinases to daytime-dependent thiamine administration, with PDK3 being upregulated in the cerebral cortex in the evening, and PDK4 being induced by thiamine administration in the morning [31]. Both thiamine and oxythiamine induced changes in rat locomotory behavior (latency in the open field test) and affected ECG parameters to a lesser extent (Figure 3B,C and [31]).

The stronger effects of oxythiamine on the brain are also in accordance with classic studies on thiamine deficiency and both oxythiamine and pyrithiamine administrations, where neurological manifestations occurred without any cardiological symptoms [4,17,18,24]. Finally, various mutations in genes encoding ThDP-dependent enzymes as well as thiamine pyrophosphokinase and thiamine or ThDP transporters in human patients or knockout animals also often induce stronger metabolic phenotypes in the nervous tissues compared to the heart [38,39,40,41].

Nevertheless, some effects of the oxythiamine-induced regulation of ThDP-dependent metabolism are specific to or more pronounced in the heart. For example, the reduction in PDHC activity and its endogenous holoenzyme percentage in the heart were much stronger than in the cerebral cortex (Figure 4B vs. 4A). Additionally, a heart-specific decrease in GSH level was observed (Figure 7B), in accordance with redox stress-driven multiple enzyme deficiency observed for pyrithiamine administration elsewhere [42]. Moreover, all three parameters (cardiac GSH level, PDHC activity and percentage of endogenous PDHC holoenzyme) demonstrated strong positive correlations with each other (Figure 8 and Supplementary Figure S4). This implies that heart-specific alterations by oxythiamine treatment, including PDHC activity, may be mediated by stronger redox imbalance upon initial downregulation of ThDP-dependent enzymes.

### 3.2. Metabolic Signature of Oxythiamine-Induced Inhibition of Cerebral ThDP-Dependent Enzymes

Our study reveals a distinct pattern of downregulated TCA cycle in cerebral cortex upon chronic oxythiamine treatment. Indeed, ThDP-dependent OGDHC belongs to the TCA cycle, and ThDP-dependent PDHC fuels the cycle with acetyl-CoA, with the activities of both enzymes in the rat cerebral cortex being reduced by oxythiamine.

First, elevated 3-hydroxybutyrate was detected (Table 1, Figure 6A,B), which is known to be a source of neuronal acetyl-CoA in the brain when the flow through PDHC is perturbed [43]. The increased 3-hydroxybutyrate may thus indicate the inability of 3-hydroxybutyrate-derived compounds, including acetyl-CoA, to be further metabolized in oxythiamine-treated animals. Indeed, acetyl-CoA catabolism is mediated by the TCA cycle where OGDHC is a rate-limiting enzyme [44,45]. Thus, even a moderate decrease in OGDHC-mediated metabolic flow may result in more substantial changes, associated with acetyl-CoA metabolism, compared to a reduced PDHC function.

Second, acetyl-CoA is known to participate in protein lysine acetylation, which is also increased by oxythiamine (Table 1, Figure 6A,B). Considering that directed cerebral PDHC downregulation by intranasal administration of its specific inhibitors previously reduced the acetylation level of proteins of the same molecular weight (~10 kDa) as here [36], the opposite changes of the cerebral metabolism in the present study (Figure 6A,B) are also mediated by the OGDHC and not PDHC downregulation.

The important role of cerebral OGDHC and, in turn, TCA cycle downregulation in mediating oxythiamine effects is also supported by the negative correlation of its activity with heart rate variability parameters, affected by oxythiamine treatment (Figure 3C and Figure 8). Furthermore, the same cerebral OGDHC activity, but not PDHC activity, shows positive correlation with blood ThDP level, itself correlating with all locomotory indicators in rats also affected by oxythiamine treatment (Figure 3B and Figure 8). Finally, similar locomotory changes were observed in our previous studies using specific OGDHC inhibitors [34] but not PDHC inhibitors [35].

Less prominent effects also support the more significant contribution of cerebral OGDHC compared with PDHC in manifestations of chronic oxythiamine treatment, such as upregulation of the pathways comprising multiple amino acids and TCA cycle intermediates (Figure 6B). Although maximal OGDHC activity levels were almost not affected (Figure 4C), oxythiamine still reduced the metabolic flow through OGDHC in the rat tissues, because of lesser concentrations of its substrate and the abundance of allosteric regulators and post-translational modifications of the enzyme [45]. Thus, even a visually subtle decrease in cerebral OGDHC activity may strongly affect various metabolic pathways in rats and their physiology.

### 3.3. Medical Roles and Potential Applications of Oxythiamine In Vivo Revealed from Its Anticoenzyme Function

Among various known thiamine antagonists [2,15,16], only oxythiamine is known to accumulate in human patients upon certain conditions, for example, severe oxidative stress and/or terminal renal failure [21]. Together with its phosphorylated derivatives mostly represented by oThDP [21], it can even be considered a physiological thiamine metabolite normally kept at a low level. However, since oThDP lacks the coenzyme function inherent to ThDP, it is commonly described as a thiamine antivitamin [21]. Despite 15-fold accumulation of plasma oxythiamine from 0.18 nM to 2.73 nM and a four-fold increase in red blood cells in oThDP from 15.9 nM to 66.1 nM upon hemodialysis of end-stage renal disease patients [21], the source of oxythiamine remains elusive. Dietary intake of acidic foods cooked at high temperatures may result in oxythiamine accumulation [21], with microbiota also proposed as a potential source of the toxic metabolite [22].

The dose of oxythiamine administered here (1.5 mg/kg) was within the range of 1.3–80 mg/kg used in similar 2–4-week-long studies in rats [11,46,47]. Noteworthy, large doses of oxythiamine (up to 80 mg/kg) had been used in combination with thiamine for 15–20 days, resulting in oThDP levels of about 250–500 nM in the rat brain and liver [46]. Although we could not measure oThDP in our model, these data illustrate that higher levels of oThDP can be induced in rat tissues, exceeding the cellular oThDP levels of patients with renal failure by about 10 times [21]. Large oxythiamine doses significantly reduced animal body weight [46], i.e., they had much stronger pathophysiological manifestations than those observed in human patients. Such negative effects on animal weight were not observed in this work.

Importantly, patients with terminal renal disease on dialysis have long been known to possess a significantly reduced red blood cell transketolase activity [21,48]. Furthermore, a case of acute Shoshin beriberi immediately after kidney transplantation in a child has been reported, with the condition rapidly resolved after intravenous thiamine administration [49]. Such renal failures often develop neuropathic complications [48,50] and possess a significantly increased risk of mortality due to cardiovascular events [22]. Described complications can now be linked to oxythiamine and oThDP [21]. Indeed, high TKT affinity to its coenzyme, with a K_m_(ThDP) value of about 40 nM [51] and oThDP accumulation up to 66.1 nM, exceeding the K_i_ value for the enzyme (40 nM [15]), can lead to the substantial TKT inactivation [21]. Due to similar K_m_(ThDP) and K_i_(oThDP) values for TKT and PDHC [2,15,20], nanomolar oThDP concentrations should also be able to inhibit the latter enzyme. These two enzymes proved to be among the main oxythiamine targets in our rat model of chronic oxythiamine exposure (Figure 2 and Figure 4). OGDHC, however, has both K_i_(oThDP) and K_m_ (ThDP) values in the micromolar range [15], but, in view of the lower ThDP content in the brain [31], the relative affinity for oThDP should be higher and the enzyme was still inhibited slightly in the oxythiamine-treated animals (Figure 4C).

In addition to being a naturally occurring thiamine (anti)metabolite, oxythiamine has been considered as a potent treatment from various types of cancer. Indeed, being a TKT inhibitor (Figure 2B), oxythiamine downregulates the pentose phosphate pathway associated with production of nucleotides and NADPH [52,53] and probably redistributes glucose oxidation into other compounds, like myo-inositol (Table 1; Figure 6A). Oxythiamine has been found to inhibit the growth and metabolism of various tumors in cell lines and animal models [19,54,55,56], and its precursor benfo-oxythiamine has proved effective as a co-therapy in a case of metastatic, refractory prostate cancer [57]. However, some cellular viability assays may indicate improved resazurin oxidoreductase activity in oxythiamine-treated cells [27]. Other thiamine antagonists, despite significant TKT downregulation, demonstrate no effects on tumor cell growth [58], implying that inhibition on other ThDP-dependent enzymes, like PDHC and OGDHC, may also mediate the anti-tumor properties of oxythiamine [15].

It is noteworthy that higher TKT expression is a marker of unfavorable prognosis in various cancer types [59,60]. Based on the fact that end-stage renal disease results in accumulation of oxythiamine [21], a hypothetical cancer-protective effect might be proposed. Evidently, other uremic toxins also accumulate upon renal failure, increasing the complexity of the potential metabolic re-arrangements. The latter hypothesis is a topic for a meta-analysis to study peculiarities and interdependencies in cancer and renal diseases. For example, despite higher incidence, some cancers occurring in patients with terminal renal disease are known to be less aggressive, leading to lower cancer-specific death rates compared to the general population, as the disease environment may hinder cancer development [61]. The latter, indeed, may be an indication of a cancer-protective role of oxythiamine accumulated in these patients at higher levels.

### 3.4. Potential Non-Coenzyme Effects of Oxythiamine In Vivo as Additional Mechanisms to Downregulate ThDP-Dependent Metabolism

Based on the downregulation of ThDP-dependent enzymes, TKT, PDHC, OGDHC (Figure 2 and Figure 4), and substantial in vitro studies (see Section 3.3), a direct inhibition of all ThDP-dependent enzymes is expected to be the main mechanism of the metabolic and physiological alterations (Figure 3, Figure 6 and Figure 7) upon oxythiamine exposure. Nevertheless, large doses of both thiamine and ThDP are known to display non-coenzyme effects. The direct binding of thiamine and derivatives regulate many targets including p53 [62], PARP1 [63], and prion protein (PrP) [64,65]. Thiamine triphosphate has been reported as a substrate for specific protein phosphorylation of 43K rapsyn [66]. Our previous works showed the effects of thiamine, its esters, and analogs on glutamate and malate dehydrogenases [37] as well as the enzyme of vitamin B6 metabolism, pyridoxal kinase [29,37]. Among those, oxythiamine was tested in vitro with the latter enzyme, with its inhibitory effects being about five times weaker than that of thiamine itself [29]. According to our knowledge, no effects of oxythiamine on glutamate and malate dehydrogenase have been described in the literature. Here, however, no changes in the activities of either enzyme upon chronic oxythiamine administration in both cerebral cortex and heart were observed; pyridoxal kinase activity was not measured, due to incompatibility of its assay protocol with the homogenization buffer used. In addition, no significant correlations of glutamate and malate dehydrogenase activities with physiological parameters were found (Figure 8). This suggests that the inhibition of TKT, PDHC, and OGDHC by oThDP upon oxythiamine supplementation rather than non-coenzyme interactions of oxythiamine and its derivatives with other targets is what caused the observed changes in rat behavior and heart regulation.

In addition, both thiamine and oxythiamine have a charged thiazolium ring (Figure 1). For both thiamine and oxythiamine, this structural feature has been suggested to be associated with definite anti-oxidant, anti-inflammatory, and anti-protein-misfolding activities, all of which should be dose-related [67]. Indeed, thiamine has been shown to act as a direct scavenger of radical species, especially negatively charged ones like peroxynitrites, forming thiochrome [67,68,69]. Nevertheless, oxythiamine should not be able to produce thiochrome [21] via similar radical scavenging. This should result in competition of rather anti-oxidant thiamine (and ThDP) and rather pro-oxidant oxythiamine (and oThDP), which can be inferred from studies on cancer cell lines, where oxythiamine and other thiamine antagonists caused ROS elevation [15] and induced p53-dependent response [28]. A decrease in cardiac GSH levels induced by chronic oxythiamine supplementation may indicate that such a pro-oxidant oxythiamine effect is observed in the heart but not in the brain (Figure 7). This tissue specificity of this pro-oxidant effect is further supported by the heart-specific positive correlations of GSH level and total PDHC activity, which were more significant in the non-treated animals (Supplementary Figure S4), with both parameters decreased in the heart more significantly than in the cerebral cortex (Figure 4A,B and Figure 7). Furthermore, multiple studies have indicated that various exposures that initiated cardiac PDHC downregulation led to cardiac redox stress [70,71]. Similar studies using brain tissues state PDHC impairment as a consequence of redox stress, not vice versa [72,73].

However, the direct antioxidant effect of thiamine and pro-oxidant effect of oxythiamine may be difficult to separate from indirect alterations in the antioxidant capacity of the cells. The latter may simply be mediated via the changes in NADPH production resulted from TKT up- or downregulation [74,75] or those mediated via OGDHC or PDHC downregulation [15,76,77,78], i.e., via coenzyme effects of ThDP and oThDP. Nevertheless, direct radical scavenging by thiamine, which should be even higher for ThDP [67,68] or its lipophilic derivatives such as benfotiamine [79], sulbutiamine [80], fursultiamine [81] or other derivatives [82], is not likely to contribute significantly to its antioxidative potential in vivo [74,79]. Similarly, the suggested thiamine effects on protein misfolding, ER stress, or potential formation of protein aggregates [83,84] could be explained solely by its coenzyme action. Nevertheless, several cases of thiamine action in Alzheimer’s disease [85,86] as well as pain release by thiamine, thiamine monophosphate, benfotiamine, and probably other thiamine forms [87,88,89,90] suggest a role for the non-coenzyme effects of thiamine and/or derivatives. Thus, non-coenzyme action of thiamine and derivatives, oxythiamine included, is well known; however, its impact is not always evident in vivo, which is in accordance with oxythiamine-mediated inhibition of TKT and other ThDP-dependent enzymes, as observed here (Figure 2B) and elsewhere [19,52].

## 4. Materials and Methods

### 4.1. Materials

All reagents were of the highest purity available and obtained from Macklin Biochemical (Shanghai, China) unless stated otherwise. Oxythiamine was obtained from Sigma-Aldrich (Steinheim, Germany), ThDP from Solarbio (Beijing, China), and most common reagents like buffers and inorganic salts, acids, and bases from Helicon (Moscow, Russia).

Reagents for TKT and ThDP assays in blood, namely, phosphopentose mixture, yeast apoTKT, triose phosphate isomerase, and glycerol-3-phosphate dehydrogenase were obtained as previously described [31,91].

Antibodies used for immunoblotting experiments are specified in Section 4.4.

### 4.2. Animal Experiments

**Figure 9:**
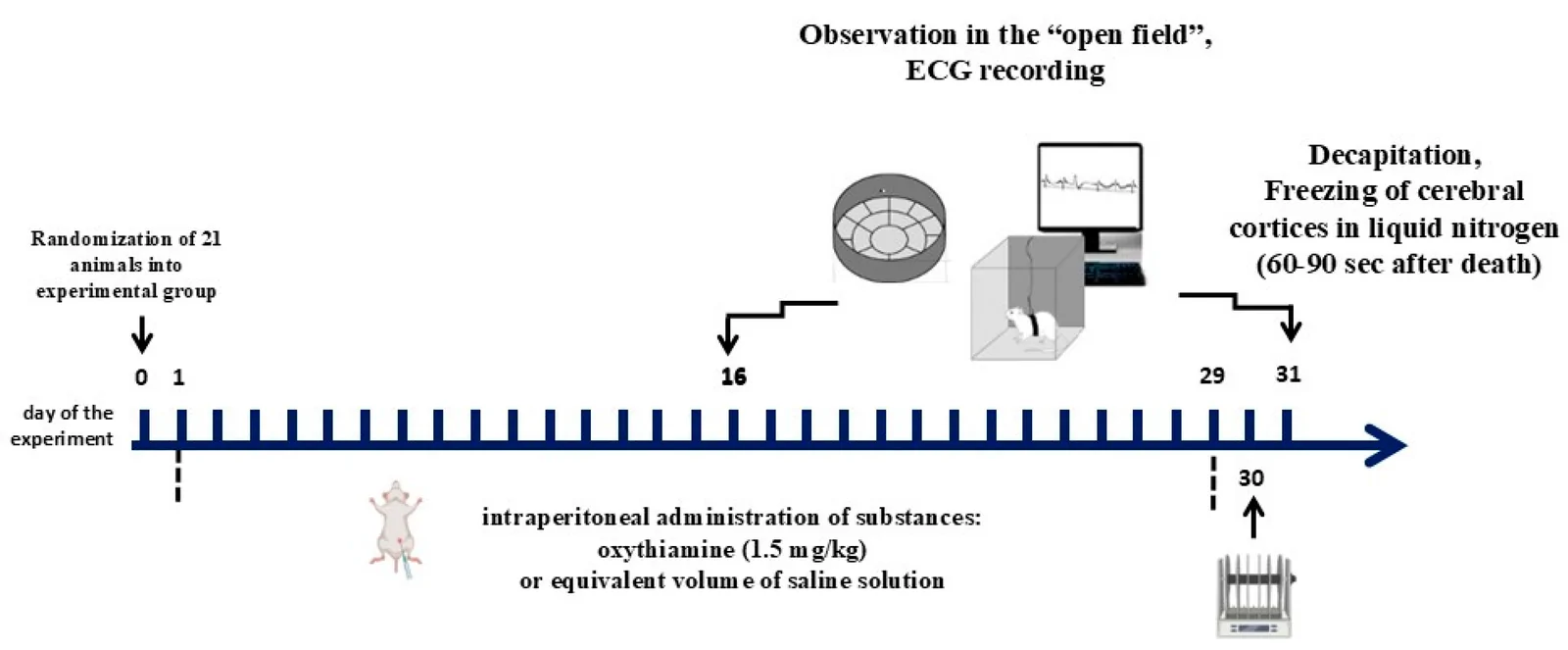
Scheme describing animal experiments. Wistar male rats (n = 21 in total) were randomly divided into four groups. Two of these groups received oxythiamine injections from day 1 to day 29, indicated by dashed lines, while saline was administered to the other two. One oxythiamine-treated and one control group (Batch 2) started the experiments three days later than the other two groups (Batch 1) since it was impossible to perform physiological tests at days 16, 30, and 31 with all rats during working hours. The rats were euthanized at day 31 and the tissues including cerebral cortices, hearts, and blood were collected and frozen in liquid nitrogen.

Twenty-one 6–7-week-old Wistar male rats obtained from the Educational and Research Center for Wildlife Rehabilitation “Chashnikovo ABS” (Moscow, Russia) were housed four to five per cage (555/4K, 580 × 375 × 200 mm) with free access to tap water and standard rodent chow (laboratorkorm.ru, Moscow, Russia), following a two-week acclimatization period. The rats were randomly allocated to two batches, each containing two experimental groups. Female rats were not included as the study was not aimed at identifying sex differences, and previous experiments also included male rats [47]. Randomization was applied using a body-weight-based procedure to ensure comparable initial weight distribution. Rats were ranked by body weight and then assigned alternately to the two batches. The same procedure was applied for allocation to the control and experimental groups. The second batch started experiments three days after the first one. Animals in the experimental group (n = 5 for the first batch, n = 6 for the second) received intraperitoneal injections of oxythiamine at a dose of 1.5 mg/kg for 29 days, while control group (n = 5 both for the first and second batches) received an equivalent volume of saline solution (Figure 9). The injections were made daily between 10:00 and 11:00 a.m. to minimize circadian-dependent variability in thiamine metabolism. No mortality was observed in any of the groups. Body weight at the beginning of the experiment was 162 ± 11 g in the control group and 162 ± 9 g in the experimental group, and at the end of the experiment, body weight was 276 ± 14 g and 277 ± 10 g, respectively.

Physical endurance was evaluated using a rotarod apparatus (Neurobotics LLC, Russia) on day 30. Animal testing was conducted in two stages. During the first stage, animals were acclimated to the apparatus, with several placements in individual compartments until stable maintenance on the rotating rod was achieved at an initial drum rotation speed of 4 rpm. During the second stage (final testing), initial and final speed settings were set at 4 and 40 rpm, respectively, with a maximum exposure time of 300 s, providing constant acceleration from 4 to 40 rpm over 5 min. Fall latency and distance travelled were recorded and averaged upon three repeated tests; animals were allowed to rest in their home cages for 30 min to prevent fatigue between trials.

Open field testing together with ECG recordings were performed on days 16 and 31. Spontaneous behavior in a novel environment was assessed using the open field test (OpenScience, Moscow, Russia). In accordance with established protocols [92], animals were tested for three minutes under silent conditions illuminated by a 15 W red lamp. The 3 min duration was selected based on prior observations in this rat model, in which the most active behaviors (line crossings, central entries, rearing) occurred during the first 3 min and then sharply declined. This interval therefore captured the most informative phase of the initial novelty response and was sufficient to detect the effects of oxythiamine treatment. Locomotor activity was quantified as total number of line crossings as well as the numbers of central square entries and returns to area walls. Exploratory activity was measured by counting rearing episodes. Anxiety-like behavior was evaluated based on the number of defecation bouts, duration of freezing, and the duration and frequency of grooming episodes.

ECG recordings were performed for 3 min using non-invasive electrodes as previously described [92]. The animals were pre-adapted to the recording setup for 10 min. During this period, each animal was placed in a loose-fitting jacket and allowed to move freely within an experimental chamber. ECG recording was initiated only after the animal had settled and displayed a stable baseline heart rate. This approach avoids the confounding effects of anesthesia and handling stress on autonomic parameters. The 3 min recordings reliably detect changes in autonomic modulation [93]. The balance of autonomic heart regulation was assessed through the following ECG parameters: mean R-R interval (ms), standard deviation of R-R intervals (SD, ms), range of R-R interval values (dX, ms), root mean square of successive differences between normal heartbeats (RMSSD, ms), and stress index (SI, arbitrary units).

The experimenters were blinded to group allocation during behavioral testing, ECG recording, and sample collection. Group assignment was revealed only during data analysis. The same approach was used for other measured parameters with an exception for immunoblotting.

### 4.3. Tissue Extraction for Protein and Metabolite Assays

After physiological assessment on the 31st day of the experiment, the animals were decapitated, their brains were excised with cerebral cortices rapidly separated on ice and frozen in liquid nitrogen within 90 s from decapitation. No anesthesia or sedation was administered prior to decapitation, since anesthetics are known to induce strong metabolic changes in the brain; this protocol was approved by the local bioethics committee and is consistent with our previous study [29]. Simultaneously, the whole hearts were rapidly excised by another professional and also frozen in liquid nitrogen. Blood was collected from jugular veins during decapitation into heparin-containing tubes and also frozen. All samples were stored at −70 °C until analysis.

After several weeks of storage at −70 °C, the cortices and hearts were homogenized in the 50 mM MOPS pH 7.0, containing 2.7 mM EDTA, 20% glycerol and protease inhibitors, as described [34,94]. Before the enzyme assays, the mitochondrial and other-membrane associated enzymes were extracted by sonication of homogenate samples in a Bioruptor device (Diagenode, Liege, Belgium) followed by the addition of one third volume of 40 mM Tris-HCl buffer, pH 7.4, containing 600 mM NaCl, 4 mM EDTA, 1% sodium deoxycholate, and 4% NP-40. Blood samples stored at −70 °C were also sonicated before the assays, but the solubilization buffer was not added.

### 4.4. Enzyme Assays and Immunoblotting

The enzyme activities were measured in sonicated and detergent-treated brain or heart homogenates, or in case of TKT, in sonicated blood samples, as described previously [31]. Briefly, PDHC was measured in the medium containing 50 mM KH_2_PO_4_, pH 7.5, 3.2 mM L-carnitine, 1 mM dithiothreitol, 1 mM MgCl_2_, 50 µM coenzyme A, 2.5 mM NAD^+^, 5 mM pyruvate, and none or 0.2 mM ThDP, using fluorometric detection of NADH formation rate in CLARIOStar Plus plate reader (BMG LABTECH, Ortenberg, Germany). To omit the known interference from the lactate dehydrogenase consuming NADH and pyruvate, the assay medium was supplemented 25 mM oxamate inhibiting the enzyme [95].

OGDHC was assayed similarly in the medium containing 50 mM MOPS, pH 7.0, 1 mM dithiothreitol, 1 mM MgCl_2_, 1 mM CaCl_2_, 50 µM coenzyme A, 2.5 mM NAD^+^, 2 mM 2-oxoglutarate, and none or 1 mM ThDP. Percentages of endogenous holoenzyme fractions (%holo) of PDHC and OGDHC were calculated as ratios between their activities without and with ThDP added to the medium, multiplied by 100%. Percentages of the corresponding apoenzyme fractions (%apo) were calculated as 100—%holo.

Glutamate and malate dehydrogenases were assayed using spectrophotometric detection of NADH consumption rate in media containing 100 mM Tris-HCl, pH 7.5, supplemented with either 2.5 mM 2-oxoglutarate, 0.2 mM NADH and 50 mM NH_4_Cl or 0.3 mM oxaloacetate and 0.14 mM NADH, respectively, using a Sunrise plate reader (Tecan, Vienna, Austria).

Blood TKT activity was determined based on NADH consumption rate in coupled reaction using spectrophotometric detection in CLARIOStar Plus. The assay media contained 50 mM glycylglycine, pH 7.6, 2.5 mM MgCl_2_, 1 mM NADH, 13.5 U/mL triosephosphate isomerase, 0.9 U/mL glycerol-3-phosphate dehydrogenase, and none or 0.2 mM ThDP. The reaction was initiated by adding 3 mg/mL of phosphopentose mixture after the preincubation of blood samples in the assay media until NADH consumption rate becomes constant. Percentages of the holoenzyme and apoenzyme fractions were calculated as described for PDHC and OGDHC.

All enzyme activities were blank-corrected based on the NADH accumulation/consumption rates in assay media without phosphopentoses (TKT) or 2-oxo acid substrates (pyruvate for PDHC, 2-oxoglutarate for OGDHC and glutamate dehydrogenase, oxaloacetate for malate dehydrogenase) and normalized per mL of blood or per g of tissue fresh weight.

Total protein acetylation and protein expression of mitochondrial deacetylases was estimated using immunoblotting and antibodies for acetylated lysine residues, SIRT3 and SIRT5 proteins. To prepare samples for immunoblotting cerebral cortex homogenates were mixed with Laemmli buffer and incubated at 95 °C for 5 min. Proteins were separated by SDS-PAGE and visualized using 2,2,2-trichloroethanol staining followed by the transfer on PVDF membranes. Subsequently, membranes were blocked for 1 h in Tris-buffered saline with 0.1% Tween-20 (TBST) containing 5% BSA followed by the incubated for 1.5 h with primary and 45 min with secondary antibodies (both diluted in TBST containing 0.5% BSA) for 1.5 h, followed by a 45 min incubation with secondary antibody. The following primary antibodies were used: rabbit anti-acetylated-lysine (Cell Signaling Technology (CST), Danvers, MA, USA, #9814, 1:2000), rabbit anti-SIRT3 (CST #5490, 1:5000), rabbit anti-SIRT5 (CST #8782, 1:5000). The secondary antibody was HRP-linked goat anti-rabbit IgG (Imtek, Moscow, Russia, #P-GAR Iss, 1:5000). Chemiluminescence and fluorescence signals were detected using ChemiDoc MP Imager (Bio-Rad, Hercules, CA, USA) and processed in Image Lab software v. 6.0.1 (Bio-Rad, Hercules, CA, USA). For quantification, the band intensities (peak areas) were normalized to the total protein signal from the corresponding lane in the gel. The band intensities from different membranes were compared across all the membranes after the normalization on the control samples repeated on the membranes. Raw images of antibody-stained membranes and trichloroethanol-stained gels are shown in Supplementary Figure S5.

### 4.5. Metabolite Assays

ThDP from whole blood was extracted by heating and measured spectrophotometrically based on activation of TKT reaction catalyzed by yeast TKT apoenzyme [89]. We confirmed that the method conditions were applicable not only for the 0.1–0.4 µM ThDP determined (Figure 2A), but that up to 2 µM ThDP could be effectively determined due to the excess of TKT. Taking into account the tight binding of both ThDP and oThDP to the first active site of TKT apoenzyme [96], and the oThDP levels not exceeding 0.5 µM concentration even at much higher doses of oxythiamine [46], the presence of oThDP could not influence the measurement of ThDP significantly. Blood glucose concentration was measured using a portable Accu-Chek glucometer (Roche Diagnostics, Rotkreuz, Switzerland) according to the manufacturer’s instructions.

Metabolites from cerebral cortex and hearts were quantified in methanol or methanol-acetic acid extracts prepared as described in [97]. Briefly, tissue samples were homogenized in 8 volumes of ice-cold methanol. The proteins were then precipitated by a 20 min centrifugation at 18,000× *g* and 4 °C, which followed the addition of 1.5 volumes of 0.2% acetic acid and 30 min incubation on ice for the determination of GSH and GSSG. Both types of extracts were stored at −80 °C until analyses.

GSH level was measured using colorimetric assay with 5,5′-dithiobis-(2-nitrobenzoic acid (DTNB) [97]. GSSG level was measured using fluorometric assay with orto-phthalaldehyde [34]. Other metabolites from the rat cerebral cortex were analyzed using NMR. For this, 600 μL of methanol extract was mixed with 1000 μL of chloroform and 400 μL of 0.9% NaCl solution in water. The mixture was incubated at −20 °C for 20 min followed by 20 min centrifugation at 14,000× *g* and 4 °C. The resulting supernatant was collected into an Eppendorf tube, evaporated under a nitrogen stream, and subsequently stored at −80 °C. Before NMR analysis, the dried residue was reconstituted in 500 μL of deuterated phosphate buffer (150 mM, pH 7.15) containing 2 mM sodium azide as a preservative and 0.1 mM DSS-d6 (Sigma-Aldrich, Steinheim, Germany) as an internal standard. Following a short centrifugation (10 min at room temperature), the samples were transferred into standard 5 mm NMR tubes. NMR spectra were recorded at 25 °C on a 700 MHz Bruker AVANCE Neo spectrometer fitted with a Prodigy triple-resonance cryoprobe (Bruker, Billerica, MA, USA), employing the noesypr1d pulse sequence (131,072 data points, 4 dummy scans, 800 scans, spectral width of 19.8364 ppm, acquisition time of 4.7 s, relaxation delay of 3.0 s). Spectral processing, metabolite assignment, and quantification based on the DSS peak area were carried out using Chenomx NMR Suite v9.0 (Chenomx Inc., Edmonton, AB, Canada). All quantified metabolites are presented in Supplementary Table S1.

### 4.6. Data Processing and Statistics

For multivariate statistical analyses of NMR metabolomics data, values for all metabolites, together with enzyme activities and immunoblotting band intensities were batch-corrected, i.e., normalized to the median value in the control group of the same batch (see Section 4.2). When analyzed separately, both biochemical and physiological parameters were presented as uncorrected data.

Outliers for multivariate analyses were detected using IQR test in rstatix package (v. 0.7.3) in R (version 4.5.2, used within RStudio (v. 2026.01.0, Posit Software, PBC, Boston, MA, USA) shell) applied to the batch-corrected values. One sample from control group of Batch 1 and was excluded, as levels of its multiple (≥5) metabolites were extreme outliers.

For single-parameter analyses, an iterative Grubbs test in GraphPad Prism v. 10.0 (GraphPad Software, LLC, San Diego, CA, USA) was applied to the uncorrected values; the detected outliers were similarly excluded from statistical analyses.

Multivariate analyses for metabolite and other biochemical parameters, i.e., PCA and PLS-DA, and statistical validation of the models, including repeated three-fold partition and permutation testing, were performed using the mixOmics package (v. 6.32.0) in R [98]. Features with VIP score > 1.5 were retained for further exploratory visualization and interpretation.

Significances of differences between mean batch-normalized (metabolite levels) or raw (physiological and other biochemical parameters) values of control and treated samples were analyzed using unpaired *t*-test (for normally distributed values in two groups according to Shapiro–Wilk test), Mann–Whitney U-test (for non-normally distributed values in two groups), or ANOVA with repeated measurements and Sidak post hoc test (four paired groups). Some of the tests required grouping of metabolites using classification based on KEGG pathways by KEGGREST (v. 1.48.1) R package (https://github.com/Bioconductor/KEGGREST (accessed on 30 January 2026)) or manual grouping (Supplementary Table S1). For enrichment analysis, significance of changes within metabolic pathways were analyzed for averages of z-scored parameters within a pathway group for each sample.

Correlations between the parameters were estimated using Spearman’s correlation coefficients. For calculation of group-corrected Spearman correlations, ranks in each pair of parameters across all animals were corrected by subtracting group mean rank; *p*-values of correlations were determined using a *t*-test with n-3 degrees of freedom, instead of the default (n-2), where n is the number of paired ranks.

Differences and correlations with *p* ≤ 0.05 were considered significant, with 0.05 < *p* ≤ 0.1—trends to a significance.

## 5. Conclusions

Oxythiamine and oThDP are present in the blood of all humans, and pathophysiological conditions stimulating their accumulation have been revealed recently. Thus, studying the tissue-specific effects of oxythiamine, primarily on the brain and heart, has become even more relevant. Our model of chronic exposure of rats to oxythiamine demonstrates that relatively minor changes in the cerebral metabolic network were associated with substantial changes in animal locomotory activity, anxiety-like behavioral changes, and heart rate variability. Of those changes, cerebral pyruvate dehydrogenase activity was one of the most downregulated parameters, although alterations in the metabolic network also suggest a substantial role for the inhibition of 2-oxoglutarate dehydrogenase complex. Tissue-specific effects of oxythiamine exposure on cardiac metabolism included much stronger inhibition of pyruvate dehydrogenase, together with stronger reduction in glutathione level as an indicator of oxidative stress. Our findings thus provide a mechanistic foundation for understanding tissue-specific effects of chronic thiamine deficiency and highlight the potential for oxythiamine as a tool for research in metabolic engineering.

## Figures and Tables

**Table 1:** Changes in metabolic parameters in rat cerebral cortex induced by chronic oxythiamine treatment. Significance of changes are determined with *t*-test and *p*-values were adjusted by FDR for each metabolic feature with overall VIP score > 1.5. Asterisks * indicate differences between control and oxythiamine-treated groups according to *t*-test (*p* ≤ 0.05), # indicate trends (0.05 < *p* ≤ 0.1).

Metabolite/Enzyme	VIP Scores			Fold Change
	Overall	Partitioned (Mean ± SD)	% of Cases with VIP > 1	
AcK 10 kDa	2.11	1.76 ± 0.43	96.4	1.43 *
PDHC + ThDP	2.02	1.69 ± 0.51	91.7	0.83 *
myo-Inositol	1.90	1.56 ± 0.39	91.9	1.08 *
OGDHC + ThDP	1.88	1.59 ± 0.38	93.8	0.94 *
3-Hydroxybutyrate	1.88	1.57 ± 0.48	87.7	1.34 #
Malate	1.73	1.45 ± 0.44	84.8	1.16 #
Methionine	1.59	1.30 ± 0.47	73.6	1.25 #
Glutamate	1.58	1.31 ± 0.46	75.5	1.06
o-Phosphocholine	1.52	1.27 ± 0.46	72.3	1.09

## Data Availability

Raw data are available from the authors upon request.

## Supplementary Materials

The following supporting information can be downloaded at: https://www.mdpi.com/article/10.3390/ijms27209000/s1.

## Abbreviations

The following abbreviations are used in this manuscript:

CST	Cell Signaling Technology
ECG	Electrocardiography
GSH	Reduced glutathione
GSSG	Oxidized glutathione, or glutathione disulfide
NMR	Nuclear magnetic resonance
OGDHC	2-oxoglutarate dehydrogenase complex, or α-ketoglutarate dehydrogenase complex
oThDP	Oxythiamine diphosphate
PCA	Principal component analysis
PDHC	Pyruvate dehydrogenase complex
PLS-DA	Partial least squares—discriminant analysis
SD	Standard deviation
RMSSD	Root mean square of successive differences
TBST	Tris-buffered saline with 0.1% Tween-20
TCA	Tricarboxylic acid
ThDP	Thiamine diphosphate, or thiamine pyrophosphate, or cocarboxylase
TKT	Transketolase
VIP	Variable importance in projection
