# Supplementary material for: Cerebral Metabolism Is More Susceptible to Oxythiamine Exposure Compared to Cardiac Metabolism
Source: Int J Mol Sci. 2026 Oct 11;27(20):9000. doi: 10.3390/ijms27209000 (PMC13704896; doi:10.3390/ijms27209000)
Supplement: Supplementary file 1 [file ijms-27-09000-s001.zip › ijms-4595671-supplementary.pdf]

International Journal of *Molecular Sciences*

MDPI

Supplementary Material

Cerebral Metabolism Is More Susceptible to Oxythiamine Exposure Compared to Cardiac Metabolism

Supplementary Figures

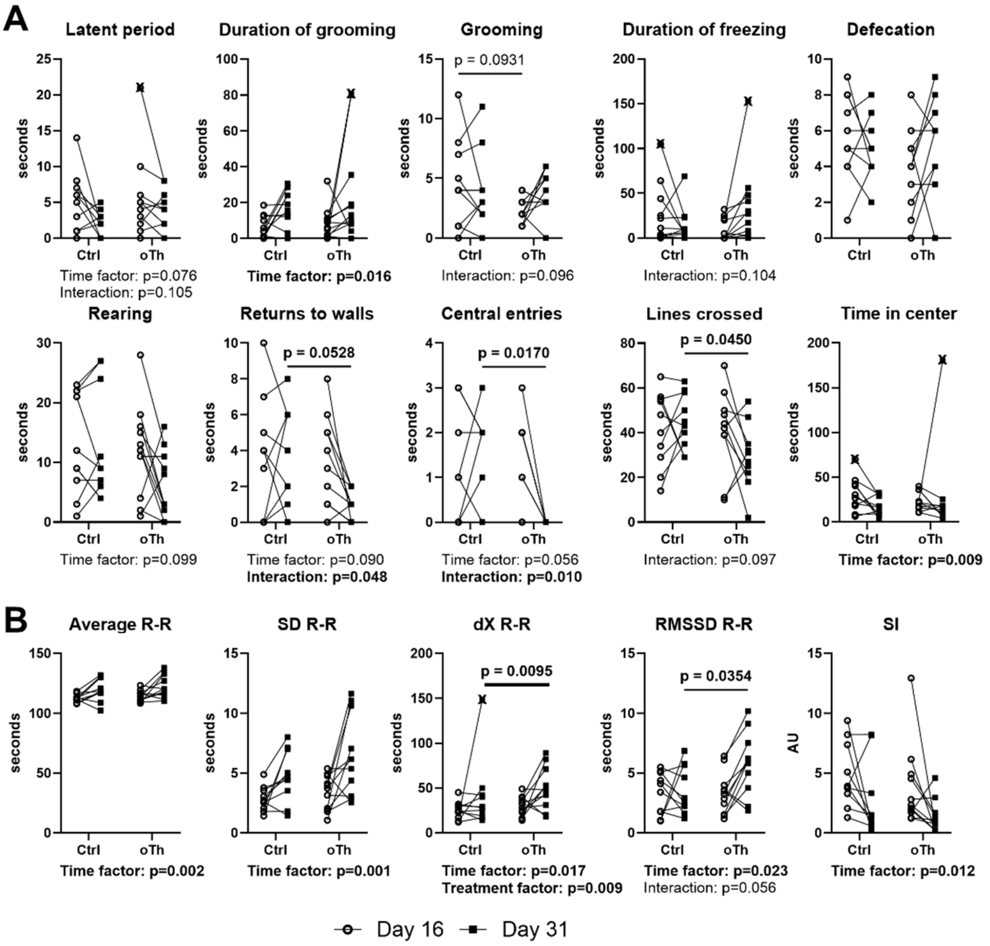

Supplementary Figure S1. Comparison of changes in the rat physiology upon 16 and 31 days of chronic oxythiamine (oTh) treatment. (A) Behavioral parameters in Open Filed Test. (B) Heart rate and its variability parameters measured by ECG. Significant differences ($p \leq 0.05$) and trends ($0.05 < p \leq 0.1$) between the control (n=10) and oTh-treated (n=11) groups evaluated using two-way ANOVA with repeated measurements are shown as p-values below (factor significances) and above (pairwise comparisons between control and oTh-treated groups according to Sidak's post hoc test) the graphs; "X" indicate outliers excluded using Iterative Grubbs' test (see Materials and Methods), in which cases mixed-model analysis was applied instead.

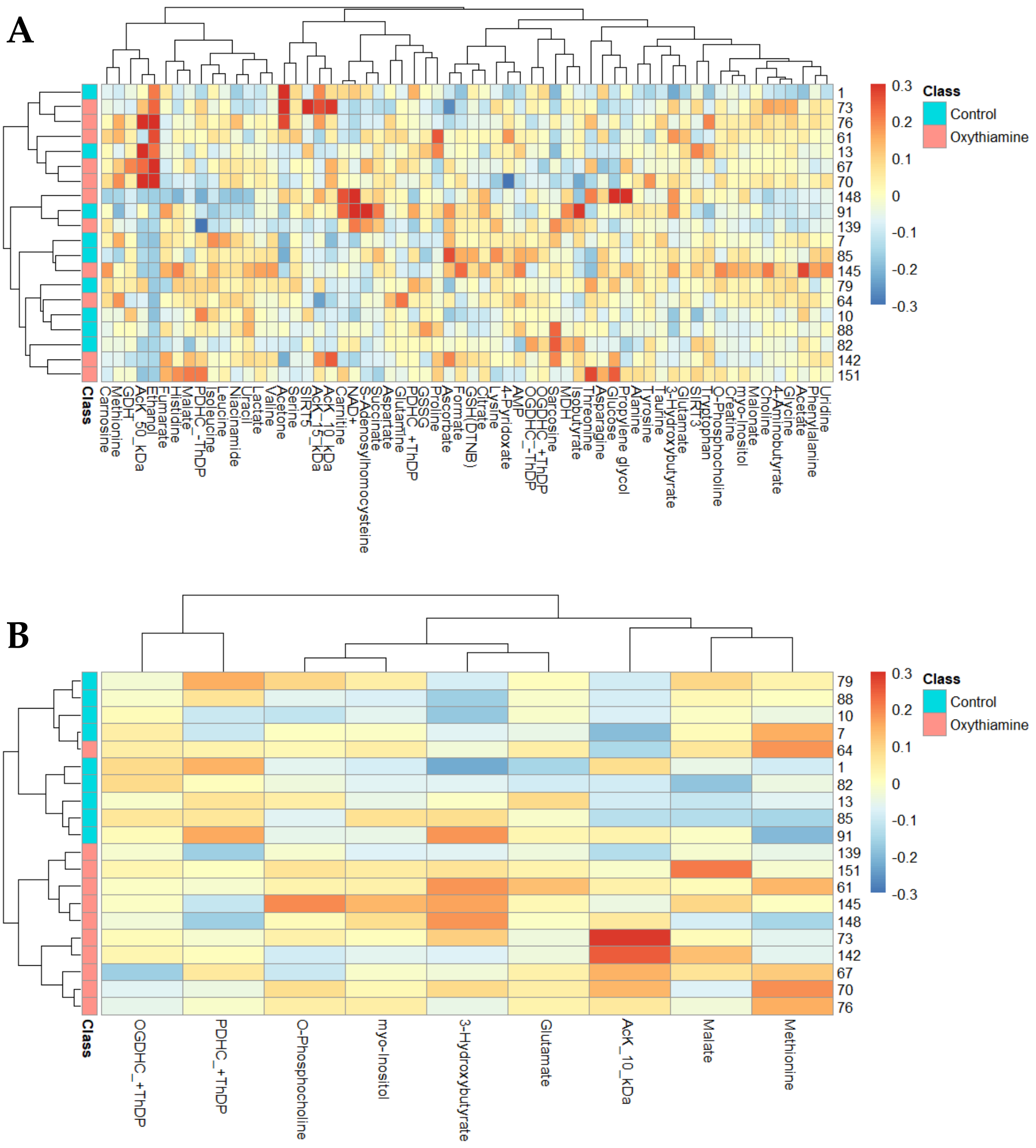

Supplementary Figure S2. Heatmap of all (A) and selected (B) biochemical parameters in the cerebral cortex of rats of control group (n = 9) and upon oxythiamine treatment (n = 11). The selected parameters (B) were chosen based on the highest (>1.5) VIP scores for the first PLS-DA component.

https://doi.org/10.3390/ijms27209000

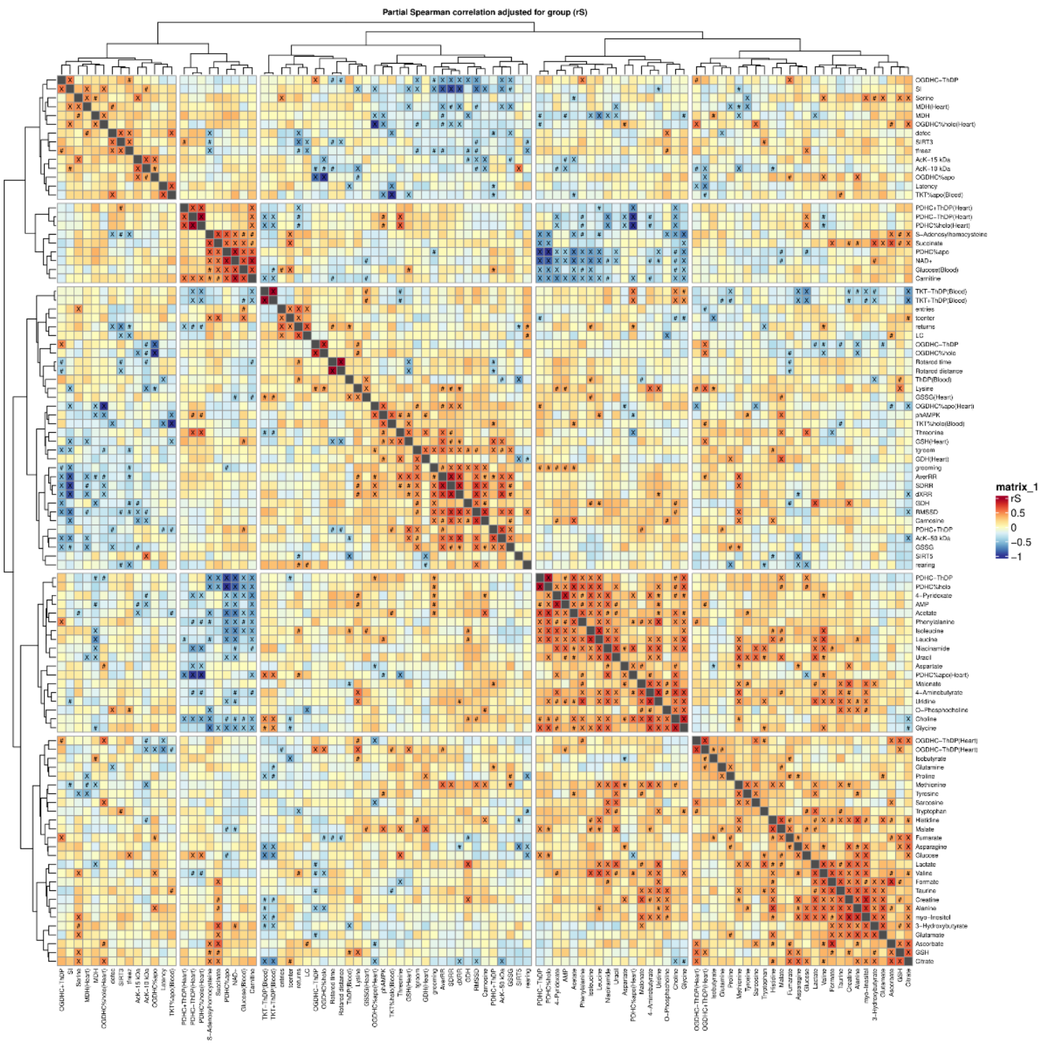

Supplementary Figure S3. Group mean-adjusted Spearman correlations of physiological vs metabolic parameters in control and oxythiamine rats combined (n = 21). Markings, abbreviations and depictions of the parameters are the same as in Figure 8 in the main text.

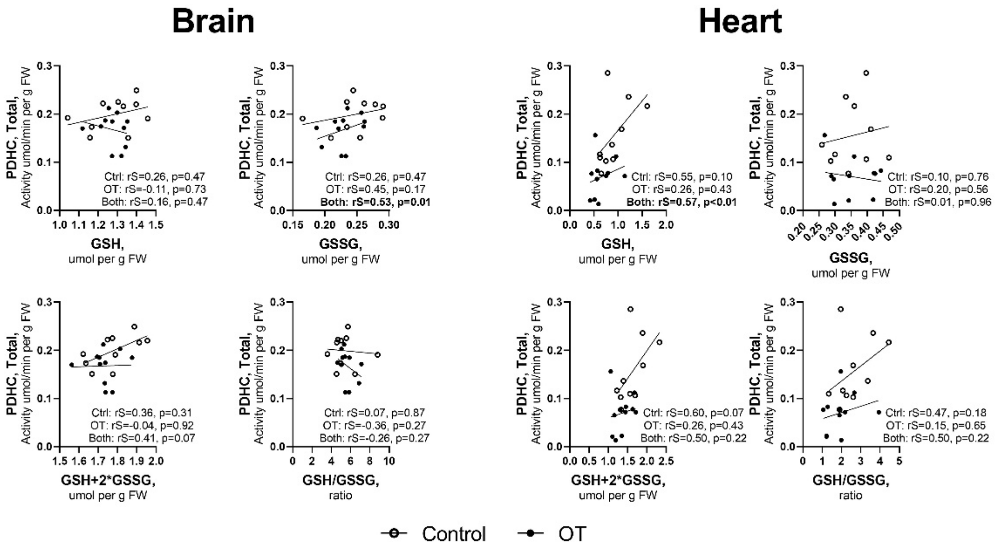

Supplementary Figure S4. Correlations between the total PDHC activity and glutathione status parameters in the brain (left panels; n = 21) and heart (right panels; n = 21) samples of control and oxythiamine-treated (OT) rats. Hollow and solid circles, approximated by linear regression, correspond to the control and OT animals, respectively. Spearman's correlation coefficients (rS) and p-values (p) for each group separately and both groups combined are shown in the graphs. Parameters of significant correlations are shown in bold.

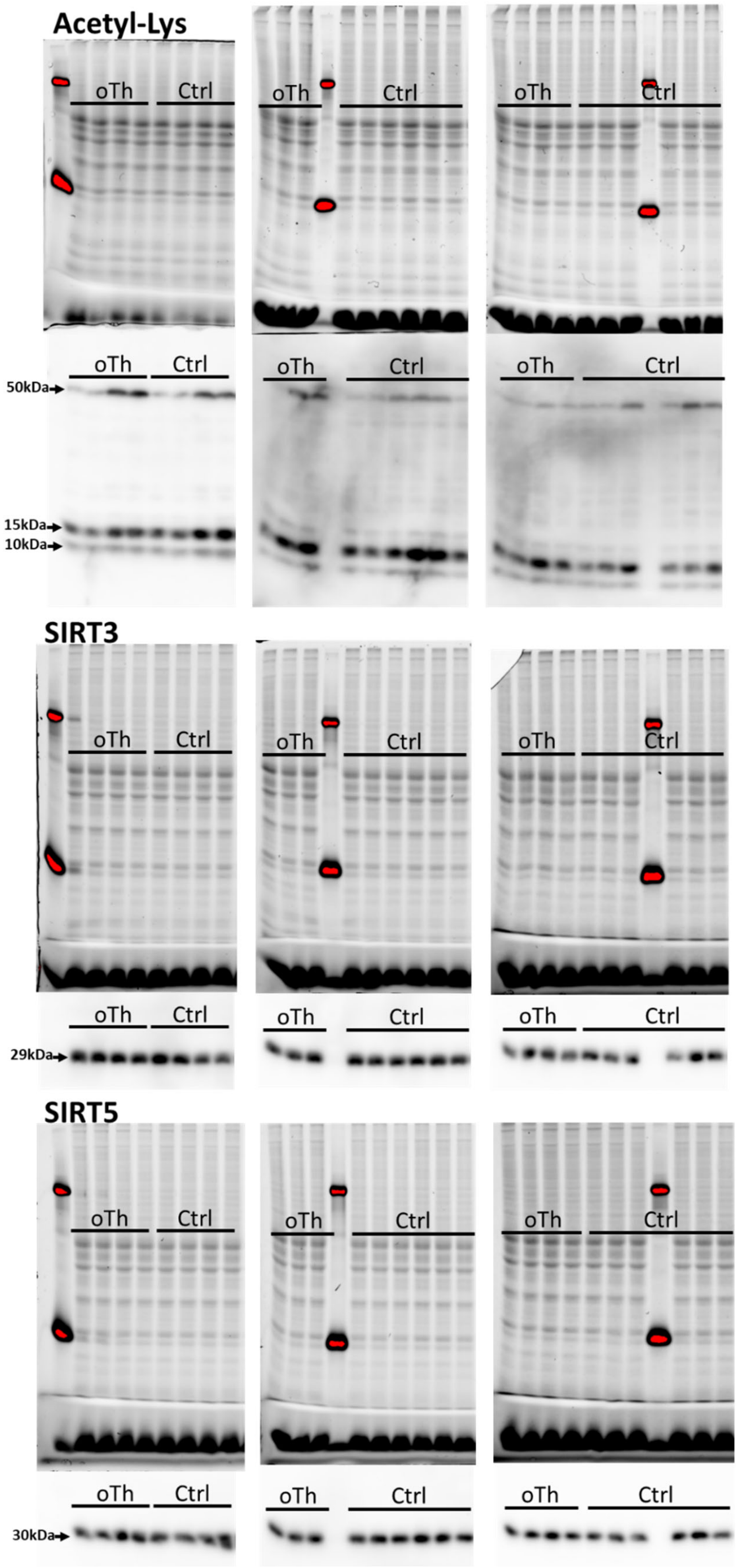

Supplementary Figure S5. Unprocessed immunoblotting images (bottom panels) showing detection of acetylated proteins, sirtuin 3 and sirtuin 5 bands, together with the corresponding trichloroethanol-stained gels (top panels) in cerebral cortex of control rats and upon oxythiamine (oTh) treatment.

https://doi.org/10.3390/ijms27209000

Supplementary Tables

Supplementary Table S1. Cerebral cortex metabolites quantified with NMR spectroscopy.

Group	Metabolites
Proteinogenic amino acids	Alanine, glycine, serine, threonine, valine, leucine, isoleucine, methionine, phenylalanine, tyrosine, tryptophan, lysine, histidine, proline, asparagine, aspartate, glutamate, glutamine
Amino acid derivatives	Choline, O-phosphocholine, S-adenosylhomocysteine, sarcosine, carnitine, carnosine, creatine, taurine, 4-aminobutyrate
Organic acids	Acetate, formate, isobutyrate, malonate, succinate, fumarate
Hydroxy acids	3-Hydroxybutyrate, lactate, citrate, malate
Sugars and derivatives	Glucose, myo-inositol
Nucleotides and precursors	AMP, NAD^+, uracil, uridine
Vitamins and derivatives	Ascorbate, 4-pyridoxate, niacinamide

Disclaimer/Publisher's Note: The statements, opinions and data contained in all publications are solely those of the individual author(s) and contributor(s) and not of MDPI and/or the editor(s). MDPI and/or the editor(s) disclaim responsibility for any injury to people or property resulting from any ideas, methods, instructions or products referred to in the content.

https://doi.org/10.3390/ijms27209000
